# Reduced neutralization of SARS-CoV-2 B.1.617 by vaccine and convalescent serum

**DOI:** 10.1016/j.cell.2021.06.020

**Published:** 2021-08-05

**Authors:** Chang Liu, Helen M. Ginn, Wanwisa Dejnirattisai, Piyada Supasa, Beibei Wang, Aekkachai Tuekprakhon, Rungtiwa Nutalai, Daming Zhou, Alexander J. Mentzer, Yuguang Zhao, Helen M.E. Duyvesteyn, César López-Camacho, Jose Slon-Campos, Thomas S. Walter, Donal Skelly, Sile Ann Johnson, Thomas G. Ritter, Chris Mason, Sue Ann Costa Clemens, Felipe Gomes Naveca, Valdinete Nascimento, Fernanda Nascimento, Cristiano Fernandes da Costa, Paola Cristina Resende, Alex Pauvolid-Correa, Marilda M. Siqueira, Christina Dold, Nigel Temperton, Tao Dong, Andrew J. Pollard, Julian C. Knight, Derrick Crook, Teresa Lambe, Elizabeth Clutterbuck, Sagida Bibi, Amy Flaxman, Mustapha Bittaye, Sandra Belij-Rammerstorfer, Sarah C. Gilbert, Tariq Malik, Miles W. Carroll, Paul Klenerman, Eleanor Barnes, Susanna J. Dunachie, Vicky Baillie, Natali Serafin, Zanele Ditse, Kelly Da Silva, Neil G. Paterson, Mark A. Williams, David R. Hall, Shabir Madhi, Marta C. Nunes, Philip Goulder, Elizabeth E. Fry, Juthathip Mongkolsapaya, Jingshan Ren, David I. Stuart, Gavin R. Screaton

**Affiliations:** 1 Wellcome Centre for Human Genetics, Nuffield Department of Medicine, University of Oxford, Oxford, UK; 2 Chinese Academy of Medical Science (CAMS) Oxford Institute (COI), University of Oxford, Oxford, UK; 3 Diamond Light Source Ltd., Harwell Science & Innovation Campus, Didcot, UK; 4 Division of Structural Biology, The Wellcome Centre for Human Genetics, Nuffield Department of Medicine, University of Oxford, Oxford, UK; 5 Oxford University Hospitals NHS Foundation Trust, Oxford, UK; 6 Peter Medawar Building for Pathogen Research, Oxford, UK; 7 Nuffield Department of Clinical Neurosciences, University of Oxford, Oxford, UK; 8 Institute of Global Health, University of Siena, Siena, Brazil; 9 Department of Paediatrics, University of Oxford, Oxford, UK; 10 Laboratório de Ecologia de Doenças Transmissíveis na Amazônia, Instituto Leônidas e Maria Deane, Fiocruz, Manaus, Amazonas, Brazil; 11 Fundação de Vigilância em Saúde do Amazonas, Manaus, Amazonas, Brazil; 12 Laboratorio de vírus respiratórios-IOC/FIOCRUZ, Rio de Janeiro, Brazil; 13 Department of Veterinary Integrative Biosciences, Texas A&M University, College Station, TX, USA; 14 NIHR Oxford Biomedical Research Centre, Oxford, UK; 15 Oxford Vaccine Group, Department of Paediatrics, University of Oxford, Oxford, UK; 16 Viral Pseudotype Unit, Medway School of Pharmacy, University of Kent and Greenwich, Chatham Maritime, Kent ME4 4TB, UK; 17 Nuffield Department of Medicine, University of Oxford, Oxford, UK; 18 MRC Human Immunology Unit, MRC Weatherall Institute of Molecular Medicine, Radcliffe Department of Medicine, University of Oxford, Oxford, UK; 19 Jenner Institute, Nuffield Department of Medicine, University of Oxford, Oxford, UK; 20 National Infection Service, Public Health England (PHE), Porton Down, Salisbury, UK; 21 Translational Gastroenterology Unit, University of Oxford, Oxford, UK; 22 Centre For Tropical Medicine and Global Health, Nuffield Department of Medicine, University of Oxford, Oxford, UK; 23 Mahidol-Oxford Tropical Medicine Research Unit, Bangkok, Thailand; 24 Department of Medicine, University of Oxford, Oxford, UK; 25 South African Medical Research Council, Vaccines and Infectious Diseases Analytics Research Unit, School of Pathology, Faculty of Health Sciences, University of the Witwatersrand, Johannesburg, South Africa; 26 Department of Science and Technology/National Research Foundation, South African Research Chair Initiative in Vaccine Preventable Diseases, Faculty of Health Sciences, University of the Witwatersrand, Johannesburg, South Africa; 27 Siriraj Center of Research Excellence in Dengue & Emerging Pathogens, Dean Office for Research, Faculty of Medicine Siriraj Hospital, Mahidol University, Thailand; 28 Instruct-ERIC, Oxford House, Parkway Court, John Smith Drive, Oxford, UK

**Keywords:** SARS-CoV-2, Receptor-binding-domain, antibody, neutralization, vaccine, escape, variant, B.1.617, structure, Delta variant

## Abstract

Severe acute respiratory syndrome coronavirus 2 (SARS-CoV-2) has undergone progressive change, with variants conferring advantage rapidly becoming dominant lineages, e.g., B.1.617. With apparent increased transmissibility, variant B.1.617.2 has contributed to the current wave of infection ravaging the Indian subcontinent and has been designated a variant of concern in the United Kingdom. Here we study the ability of monoclonal antibodies and convalescent and vaccine sera to neutralize B.1.617.1 and B.1.617.2, complement this with structural analyses of Fab/receptor binding domain (RBD) complexes, and map the antigenic space of current variants. Neutralization of both viruses is reduced compared with ancestral Wuhan-related strains, but there is no evidence of widespread antibody escape as seen with B.1.351. However, B.1.351 and P.1 sera showed markedly more reduction in neutralization of B.1.617.2, suggesting that individuals infected previously by these variants may be more susceptible to reinfection by B.1.617.2. This observation provides important new insights for immunization policy with future variant vaccines in non-immune populations.

## Highlights

• Vaccine/convalescent sera show reduced neutralization of B.1.617.1 and B.1.617.2
• Sera from B.1.351and P.1 show markedly reduced neutralization of B.1.617.2
• B.1.351, P.1, and B.1.617.2 are antigenically divergent
• Vaccines based on B.1.1.7 may protect broadly against current variants

## Graphical abstract

**Figure:**
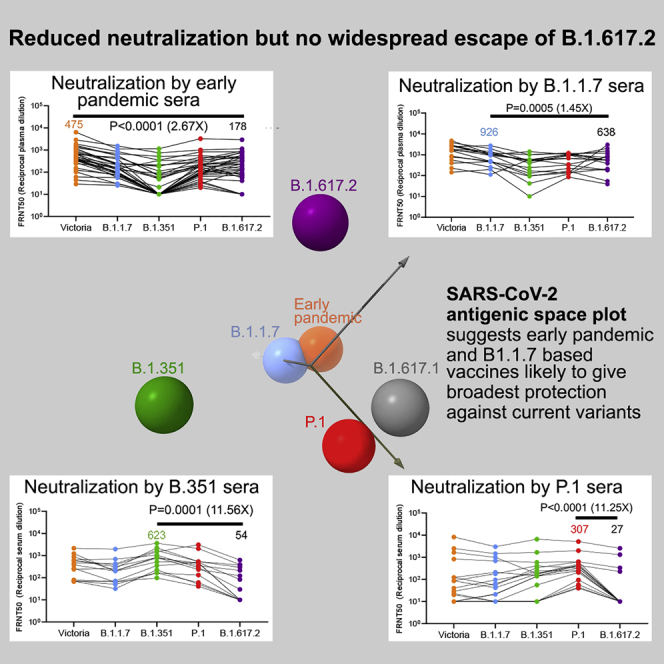

## Teaser

The B.1.617 lineage of SARS-CoV-2, especially the delta strain, which is B.1.617.2, has contributed to the wave of infection in the Indian subcontinent. Structural and serological analyses show some evidence of antibody escape, and individuals infected previously with the B.1.351 (beta) and P.1 (gamma) variants are likely more susceptible to reinfection by the delta strain. Vaccines based on B.1.1.7 (alpha) are likely to provide the broadest protection against current variants.

## Introduction

Reports of a severe acute respiratory syndrome in Wuhan, China, first appeared in December 2019. It was determined rapidly that coronavirus disease 2019 (COVID-19) was caused by infection with a novel betacoronavirus related to the severe acute respiratory syndrome (SARS) coronavirus; it was named SARS coronavirus 2 (SARS-CoV-2) (Gorbalenya et al., 2020). SARS-CoV-2 spread rapidly, leading to a global pandemic that is still accelerating and has been estimated to have led to 164 million infections and 3.4 million deaths (https://www.worldometers.info/coronavirus).

Since the first sequence of SARS-CoV-2 was deposited in early January 2020 (Lu et al., 2020), viral genome sequencing efforts have been established in a number of countries to track the evolution of the virus (COVID-19 Genomics UK (COG-UK) Consortium, 2020). Coronaviruses are large positive-strand RNA viruses, and despite some proofreading capacity (Robson et al., 2020), replication is intrinsically error prone. Progressive mutational change in the virus is therefore inevitable as it undergoes massive numbers of replicative cycles worldwide (Tegally et al., 2021). In particular, changes are anticipated as the virus adapts to its new human host.

Many thousands of mutational changes have been described across the viral genome, and although most will be detrimental or confer no advantage to the virus, some will be advantageous and be the subject of rapid natural selection (Domingo et al., 2012; Rambaut et al., 2020). Mutations could confer advantage to the virus in a number of ways, but increased transmissibility or escape from innate or acquired immune responses are two potential examples (Volz et al., 2021).

The Spike protein (S) is the major surface glycoprotein on coronaviruses. These characteristically trimeric spikes are subdivided into an N-terminal S1 domain, responsible for attachment to host cells via its receptor ACE2 (Hoffmann et al., 2020), and a C-terminal S2 domain, which is anchored in the viral membrane, cleaved from S1 following cellular attachment, and responsible for membrane fusion and cell entry. S1 consists of an N-terminal domain (NTD) followed by the receptor binding domain (RBD) which mediates binding to ACE2, burying ∼860 Å of surface area at its tip (Lan et al., 2020).

Analysis of panels of monoclonal antibodies binding to S has led to identification of a number of potently neutralizing antibodies, some of which have been developed for therapeutic and prophylactic use (Ku et al., 2021; Baum et al., 2020). Antibodies to S2 tend to be poorly neutralizing, whereas potently neutralizing antibodies generally map to S1. Most potent neutralizing antibodies bind the RBD on or closely adjacent to the ACE2-interacting surface and function to block interaction of the virus with ACE2, preventing cellular adhesion and infection (Dejnirattisai et al., 2021a; Yuan et al., 2020; Kreye et al., 2020). A second class of potently neutralizing antibodies bind to a site on the NTD called the supersite; these antibodies do not block interaction with ACE2, and their mode of neutralization is less well understood (Cerutti et al., 2021; Chi et al., 2020; Dejnirattisai et al., 2021a).

Many mutations in S have been reported, and it appears that the RBD and especially the NTD are mutational hotspots (Greaney et al., 2021). The ACE2-interacting surface of S is under intense selective pressure because changes may increase ACE2/RBD affinity, potentially increasing virus transmissibility, whereas the same changes may also reduce antibody binding to the RBD, decreasing the neutralizing potential of immune serum. In late 2020, three variants of concern were identified—B.1.1.7 in the United Kingdom, B.1.351 in South Africa, and P.1 in Brazil—that rapidly became the dominant variants locally, leading to large second waves of infection, and they continue to spread globally. These variants contain changes in the RBD: B.1.1.7 N501Y; B.1.351 N501Y, E484K, K417N, and P.1 N501Y, E484K, and K417T. These changes increase the affinity of ACE2 to RBD 7-fold for B.1.1.7 and 19-fold for B.1.351 and P.1, which may play a role in increased transmissibility. The neutralizing titers of convalescent and vaccine sera are reduced to the variants with B.1.351 of most concern, leading to a 13-fold reduction in neutralizing titers of convalescent serum, with a number of neutralizing monoclonal antibodies losing activity completely (Zhou et al., 2021; Supasa et al., 2021; Dejnirattisai et al., 2021b; Shinde et al., 2021; Madhi et al., 2021; Food and Drug Administration, 2021; Emary et al., 2021).

There are now at least 15 vaccines authorized for use in one or more countries, and these are designed to elicit antibody (and T cell) responses to S using S sequences from the original virus deposited in January 2020. Vaccines deliver S in a variety of different formats: RNA, viral vectors, recombinant protein, or inactivated virus (Krammer, 2020; Polack et al., 2020; Voysey et al., 2021; Baden et al., 2021; https://www.medscape.com/viewarticle/944933). Because the S sequence of variant viruses differs from that used for vaccination, there is concern that variant viruses may have the potential to evade antibody responses elicited by vaccination. Several studies have now shown that there is reduced vaccine efficacy against mild to moderate disease in countries where B1.351 was dominant (Zhou et al., 2021; Shinde et al., 2021; Madhi et al., 2021, Vaccines and Related Biological Products Advisory Committee, 2021), although protection against severe disease appears to be preserved. Conversely, vaccine efficacy against B.1.1.7 is maintained (Wang et al., 2021; Emary et al., 2021; Supasa et al., 2021).

In this work, we study two variant viruses, B.1.617.1 (bearing mutations L452R and E484Q in the RBD) and B.1.617.2 (bearing RBD mutations L452R and T478K), that were first reported in India at the end of 2020 but have spread globally (https://www.gisaid.org/hcov19-variants/), with B.1.617.2 causing particular concern in the United Kingdom, where it is spreading rapidly and was designated a variant of concern in May 2021. Using a panel of potent neutralizing antibodies, we show that both viruses show partial or complete escape from neutralization by some antibodies but that neutralization of most monoclonal antibodies is unaffected. Neutralization by a panel of plasma collected from convalescent individuals from the United Kingdom early in the pandemic show 4-fold and 2.7-fold reduction in neutralization titers to B.1.617.1 and B.1.617.2, respectively, compared with an early Wuhan-related strain. There are also significant reductions in neutralization titers of sera collected from recipients of the Oxford-AstraZeneca and Pfizer-BioNTech vaccines but no evidence of widespread complete escape from neutralization. We also look at the ability of sera from individuals infected with B.1.1.7, B.1.351, and P.1 to neutralize B.1.617.1 and B.1.617.2 and find that a sizeable number of sera from B.1.351 and P.1 fail to neutralize B.1.617.2. Finally, we measure the affinity of B.1.617.1 and B.1.617.2 RBDs for ACE2, showing a modest increase in affinity compared with the Wuhan RBD sequence; use structural information to identify the mechanism of escape from monoclonal antibodies; and perform a simple analysis of antigenic distances between variants to illustrate the emerging antigenic landscape of SARS-CoV-2.

## Results

### The B.1.617 lineage

**Figure 1:**
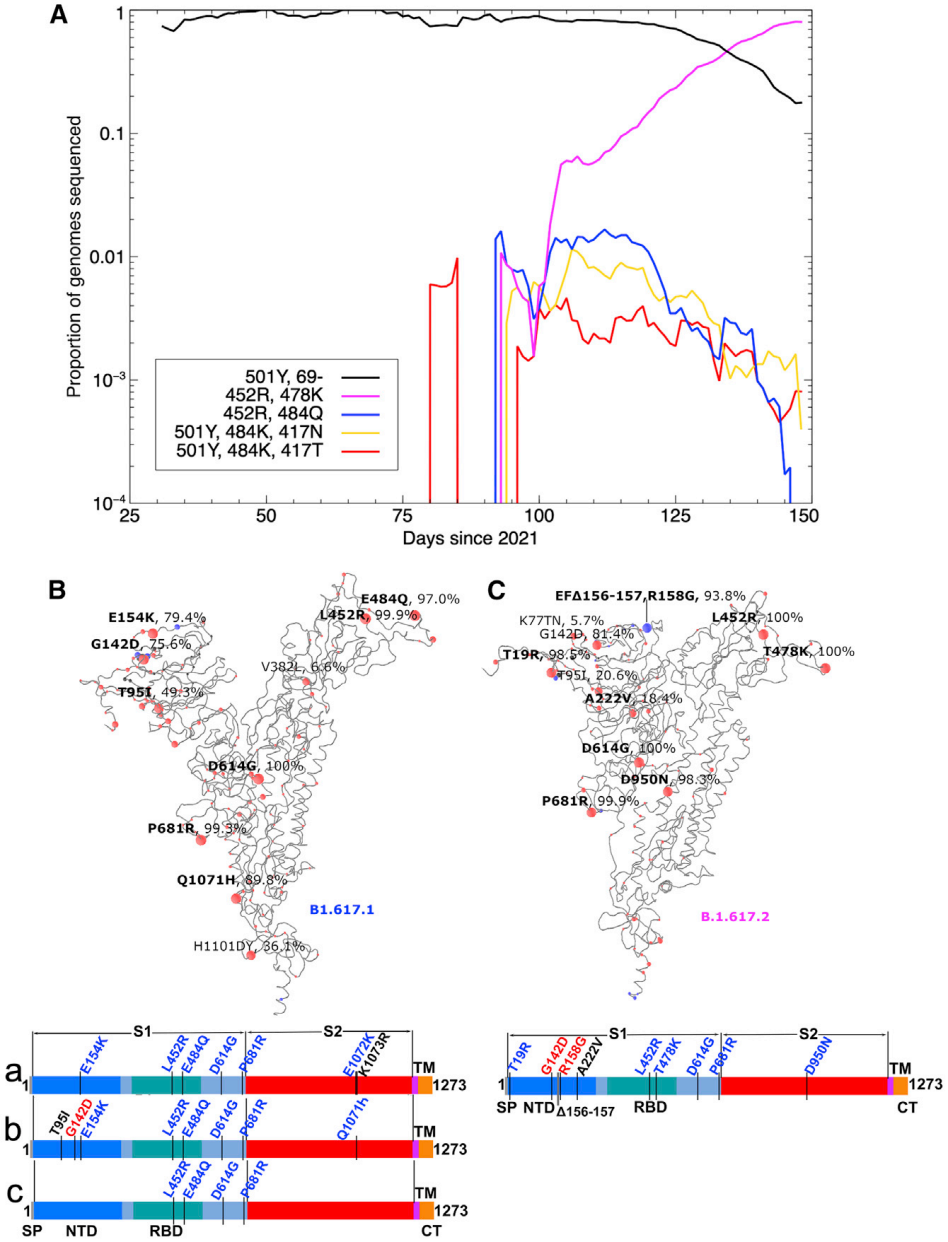
Mutational landscape of the B.1.617 lineage (A) Evolution plot showing trajectories of various mutations in the COG-UK data. Certain mutations were used to select for sequences typically belonging to a given strain: 501Y and Δ69 to select the B.1.1.7 variant; 501Y, 484K, and 417N to select the B.1.351 variant; 501Y, 484K, and 417T to select the P.1 variant; E484Q and L452R to select the B.1.617.1 variant; and T478K and L452R to select the B.1.617.2 variant. (B and C) Schematic showing the locations of amino acid substitutions in B.1.617.1 (B) and B.1.617.2 (C) relative to the ChAdOx1 SARS-CoV-2 sequence, as drawn in previous studies (Dejnirattisai et al., 2021a, 2021b; Supasa et al., 2021; Zhou et al., 2021), with all amino acid mutations above 5% explicitly labeled. Mutations colored in bold were included in the constructs used in this study for the given strain. Under the structural cartoon is a linear representation of S with changes marked for B.1.617.2 live virus, and the three subvariants of B.1.617.1 (a, b, and c) used in this study are detailed. Where there is a charge change introduced by mutations, the change is colored (red when the change makes the mutant more acidic/less basic and blue for more basic/less acidic).

There are three sublineages of B.1.617: B.1.617.1, B.1.617.2, and B.1.617.3. B.1.617.3 was the first to be identified in India, in October 2020, and is now relatively uncommon. The B.1.617.1 and B.1.617.2 variants are now found across most of the globe, including the United Kingdom, where B.1.617.2 has become the most widespread variant of concern, according to COVID-19 Genomics UK (COG-UK) data (COVID-19 Genomics UK (COG-UK) Consortium, 2020; Figure 1A). B.1.617.2 rose rapidly to dominate the sequenced genomes in the week around June 4, replacing the B.1.1.7 strain. B.1.617.1 sequences deposited into GISAID (https://www.gisaid.org/hcov19-variants/) are highly variable but contain the RBD mutations L452R and E484Q at the periphery of the ACE2-interacting surface together with P681R (which may increase furin cleavage of S1), the S2 mutation Q1071H, and up to three NTD substitutions: T95I, G142D, and E154K (Figure 1B). E484Q is a mutation at the same position as E484K seen in the B.1.351 and P.1 variants, although the change in physicochemical properties is less for the glutamine than the lysine side chain (Zhou et al., 2021; Dejnirattisai et al., 2021b). B.1.617.2 (Figure 1C) exhibits RBD mutations L452R and T478K and T19R, G142D, R158G, and A222V substitutions together with a double deletion (156-157) in the NTD and S2 substitution D950N. B.1.617.2 shares L452R and P681R with B.1.617.1, and 20% of reported sequences share T95I. L452R has also been identified in B.1.427 and B.1.429 (Deng et al., 2021), and T478K is found in B.1.1.519. Unlike B.1.617.1, B.1.617.2 contains NTD deletions, which matches a general trend of SARS-CoV-2 variants reducing the size of the NTD.

### Neutralization of B.1.617.1 and B.1.617.2 by a panel of potently neutralizing antibodies

We have previously reported generation of a large panel of 377 human monoclonal antibodies generated from individuals who had recovered from SARS-CoV-2 infection early during the pandemic (Dejnirattisai et al., 2021a). The 20 most potent neutralizing antibodies (focus reduction neutralization test 50 [FRNT50] < 0.1 μg/mL) were selected for these studies; 19 bind RBD and block interaction with ACE2, whereas the last, monoclonal antibody (mAb) 159, binds to the NTD. We used a pseudotyped lentivirus to measure neutralization of B.1.617.1 (Temperton, 2010) and a live viral isolate to measure neutralization of B.1.617.2. Neutralization of viral variants was compared with neutralization of Victoria (SARS-CoV-2/human/AUS/VIC01/2020), a Wuhan-related strain isolated early in the pandemic from Australia (Caly et al., 2020; Seemann et al., 2020).

**Figure 2:**
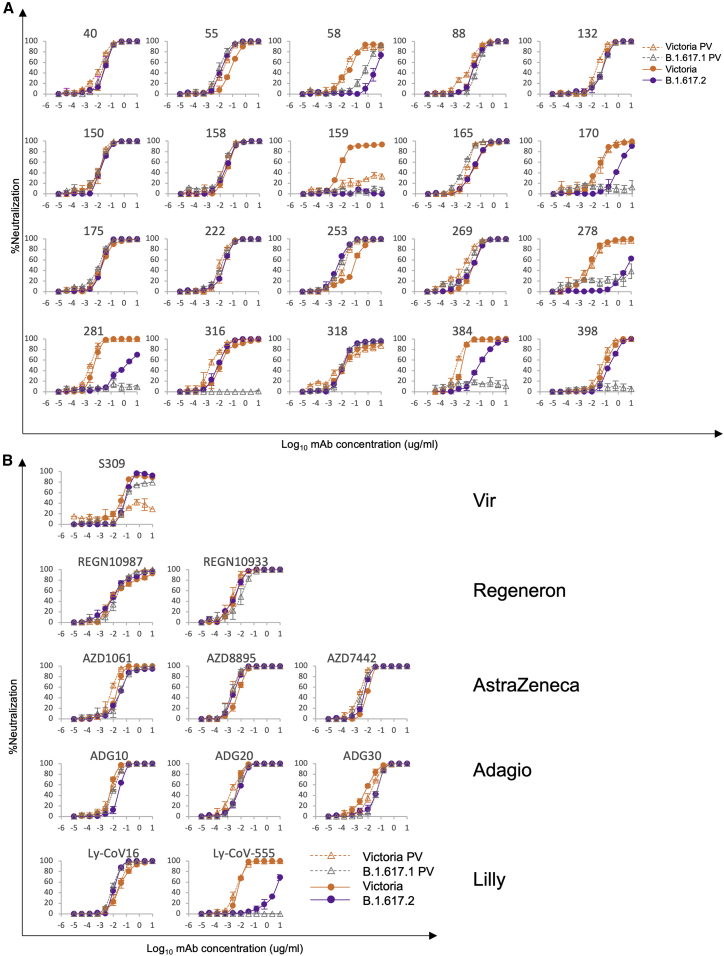
Neutralization of B.1.617.1 and B.1.617.2 by mAbs (A) Neutralization of B.1.617.1-B and B.1.617.2 by a panel of 20 potent human mAbs. Neutralization of B.1.617.1-B, as measured by pseudovirus assay, is shown as open triangles, and neutralization of B.1.617 virus, as measured by FRNT, is shown as closed circles; comparison is made with neutralization curves for Victoria, which we have generated previously (Supasa et al., 2021). Neutralization titers are reported in Table S1. (B) Equivalent plots for the Vir, Regeneron, AstraZeneca, Lilly, and Adagio antibodies.

For B.1.617.1, 8 mAbs (58, 88, 170, 278, 281, 316, 384, and 398) showed a more than 5-fold reduction in neutralization titers, with most of these showing almost complete knockout of activity (Figure 2A; Table S1). Neutralization of the B.1.617.2 virus, which shares the L452R RBD mutation with B.1.617.1, was measured using an FRNT and compared with the Victoria viral isolate. Neutralization of B.1.617.2 was reduced more than 5-fold for 6 antibodies, neutralization by NTD mAb 159 was lost completely, and neutralization by mAbs 58, 170, 278, 281, and 384 was reduced in common with neutralization of B.1.617.1, suggesting that these antibodies may share an epitope overlapping the RBD L452R substitution. Interestingly, mAb 253 showed increased neutralization of B.1.617.2.

**Figure S1:**
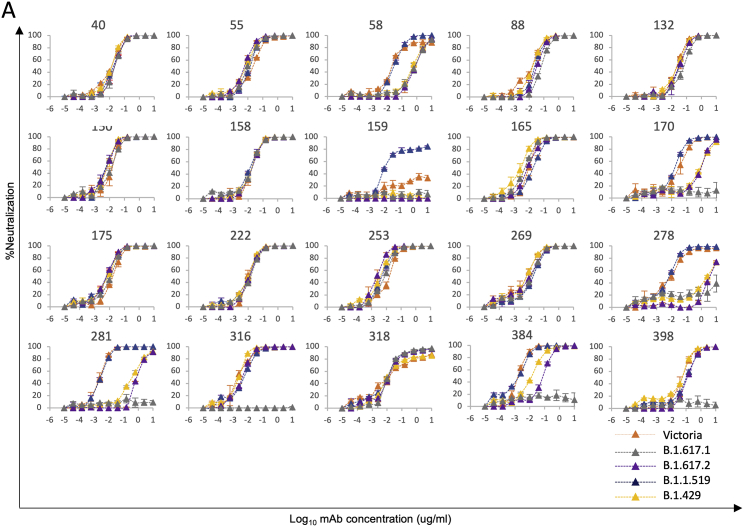
Neutralization curves of human mAbs against SARS-CoV-2 pseudotyped lentiviruses expressing full-length S of the B.1.617.1, B.1.617.2, B.1.1.519, and B.1.429 variants, related to Figure 2 FRNT50 titers are given in Table S2.

To confirm the role of the L452R RBD mutation we tested mAb neutralization with a B.1.429 pseudotyped lentivirus (containing the single L452R substitution in the RBD), which showed reduced neutralization with mAbs 58, 170, 278, 281, and 384. Finally, we performed neutralization assays on a pseudotyped lentivirus expressing B.1.1.519 S, which contains the single T478K substitution in the RBD, and saw no significant changes in neutralization (Figure S1; Table S1).

### Neutralization of B.1.617.1 and B.1.617.2 by mAbs developed for clinical use

A number of potent mAbs are being developed for clinical use, and some have received emergency use authorization (Ku et al., 2021; Baum et al., 2020; Kemp et al., 2021). We performed neutralization assays against B.1.617.1 and B.1.617.2 using antibodies S309 Vir (Pinto et al., 2020), AZD8895, and AZD1061 and the combinations AZD7442 (combining AZD1061 and AZD8895) AstraZeneca, REGN10987, and REGN10933 Regeneron; LY-CoV555 and LY-CoV16 Lilly; and ADG10, ADG20, and ADG30 from Adagio (Figure 2B; Table S1). Potent activity was maintained on B.1.617.1 and B.1.617.2, with small, up to 5-fold reductions in neutralization for some antibodies. The exceptions were LY-CoV555, which completely failed to neutralize B.1.617.1 and was reduced severely on B.1.617.2 (Greaney et al., 2021), and for unknown reasons, S309 (Pinto et al., 2020) could not neutralize the Victoria pseudotyped virus, so we could not reliably compare its activity on B.1.617.1.

### Binding of B.1.617.1 and B.1.617.2 RBD to ACE2 and mAbs

**Figure 3:**
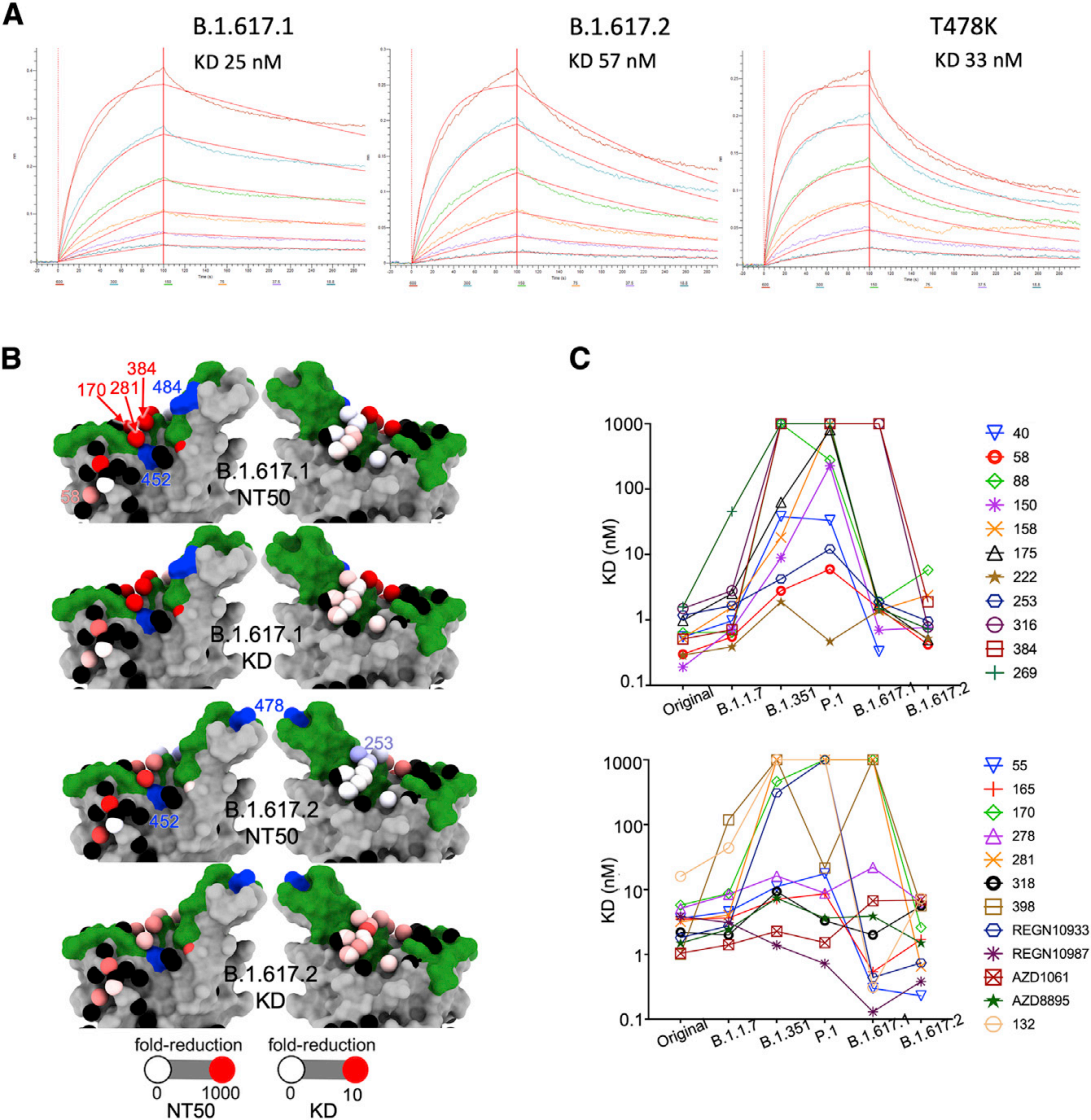
Interaction of B.1.617.1 and B.1.617.2 with ACE2 (A) BLI experiments showing binding of ACE2 to RBDs of B.1.617.1, B.1.617.2, and the T478K mutant. Experimental data for the dilution series are shown in different colors and the models as red lines. (B) Neutralization FRNT50 data (NT50) and BLI data (K_D_) mapped onto the RBD using the method described (Dejnirattisai et al., 2021a). The top two panels show the NT50 and K_D_ values for B.1.617.1, and the bottom two panels show the corresponding values for B.1.617.2. Front and back views of the RBD are shown. Spheres represent the antibody binding sites, colored according to the ratio of the values for B.1.617.1/Wuhan and B.1.617.2/Wuhan. The NT50 plots are colored white for a ratio of 1, and red for less than 0.001 (i.e., at least a 1,000-fold reduction); blue indicates that the binding is increased. For the K_D_ plots, white denotes a ratio of 1, red less than 0.1 (i.e., at least a 10-fold reduction). Black dots indicate mapped antibodies not included in this analysis, dark green indicates the RBD ACE2 binding surface, and blue shows the mutated residues in each variant. Note the strong agreement between NT50 and K_D._ All relevant data are shown in Table S1. (C) K_D_s of RBD/mAbs interactions, measured by BLI for RBDs of Victoria (original), B.1.1.7, B.1.351, P.1, B.1.617.1 and B.1.617.261 (left to right).

To understand the contribution of interactions at the RBD to the properties of the two variants, we analyzed interactions of variant RBDs with ACE2 and the panel of neutralizing antibodies using biolayer interferometry (BLI). The results for ACE2 (Figure 3A) show that the B.1.617.1 and B.1.617.2 double mutations (L452R and E484Q; L452R and T478K) show perhaps a modest increase in affinity for ACE2 (25 and 57 nM, respectively) compared with Victoria RBD (75 nM). B.1.1.519 (T478K) has a similar K_D_ (33 nM), suggesting that L452R does not significantly alter affinity for ACE2.

The results for antibody binding to RBDs mirror the neutralization results for B.1.617.1 and B.1.617.2 (Figures 3B and 3C). As expected, the affected antibodies are proximal to the mutation sites. The most affected antibodies are especially in the top and front in the neck epitope (nomenclature of Dejnirattisai et al., 2021a), with a small effect on some antibodies belonging to the right flank epitope. The reasoning presented above for assignment of individual mutations to effects on antibody potency is consistent with the site of antibody attachment.

### Structural solution for antibody escape

**Figure 4:**
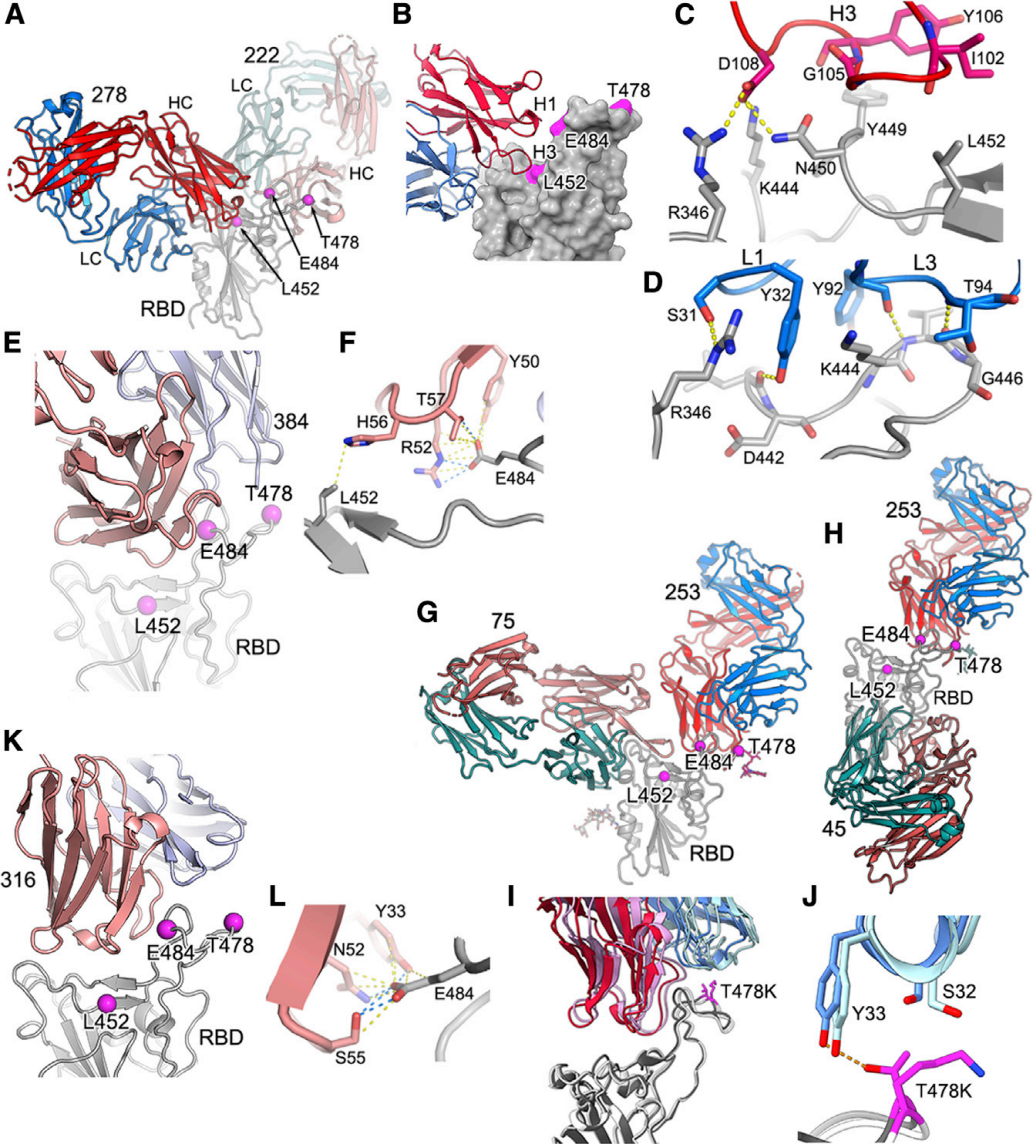
Crystal structures of RBD-Fab complexes and mechanism of reduced antibody potency to B.1.617 variants (A) Cartoon depiction of the ternary complex of Wuhan RBD (gray, magenta balls represent the mutations in the B.1.617 lineage, and this representation is also used in other panels) with antibody 278 (light chain, blue; heavy chain, red) and antibody 222 (light chain, pale blue; heavy chain, pink), which was used as a crystallization chaperone. The heavy chain of antibody 278 binds to an epitope comprising residue 452, explaining its reduced ability to neutralize B.1.617.1 and B.1.617.2. (B) Simplification of (A), showing CDR loop H3 from antibody 278 (HC, red; LC, blue) interacting with residue 452 on the Wuhan RBD, depicted as a gray surface (the B.1.617 lineage mutations are highlighted in magenta). (C and D) Specifics of antibody 278 interaction. (C) Residue D108 of H3 forms salt bridges with R346, K444 and a hydrogen bond to N450. L452R would sterically inhibit binding. (D) L1 hydrogen bonds via S31 to R346 of the RBD, and Y32 hydrogen bonds to the carbonyl of D442. L3 forms backbone hydrogen bond interactions between Y92 and K444, T94, and G446. (E and F) The binding mode of Fab 384 (E) and its interactions with L452 and E484 of the RBD (F) (PDB: 7BEP). (G) Cartoon depiction of the ternary complex of antibody 253 (HC, red [sugar shown as red sticks]; LC, blue) with mutant L452R RBD (gray, with sugar shown as sticks) with antibody 75 (HC, pink; LC, green) used as a crystallization chaperone. Antibody 253 makes no contact with R452, in line with no observed loss of neutralization. (H) Cartoon depiction of the ternary complex of antibody 253 (HC, red; LC, blue; sugar, red sticks) with T478K RBD (gray) and antibody 45 (HC, pink; LC, green) as a crystallization chaperone. (I and J) Close ups showing 253 interacting with residue 478 in the two mutant RBDs, revealing a modest shift in the binding pose of 253. The L452R mutant RBD is shown in dark gray, with antibody 253 in crimson (HC) and blue (LC), and the T478K RBD is shown in white, with 253 in pink (HC) and pale blue (LC). The Thr and Lysine at 478 are shown as magenta sticks (I). In the T478K mutant RBD, the lysine folds back away from the antibody (J). (K and L) The binding mode of Fab 316 to the RBD (K) and its interactions with E484 of the RBD (L) (PDB: 7BEH).

**Figure S2:**
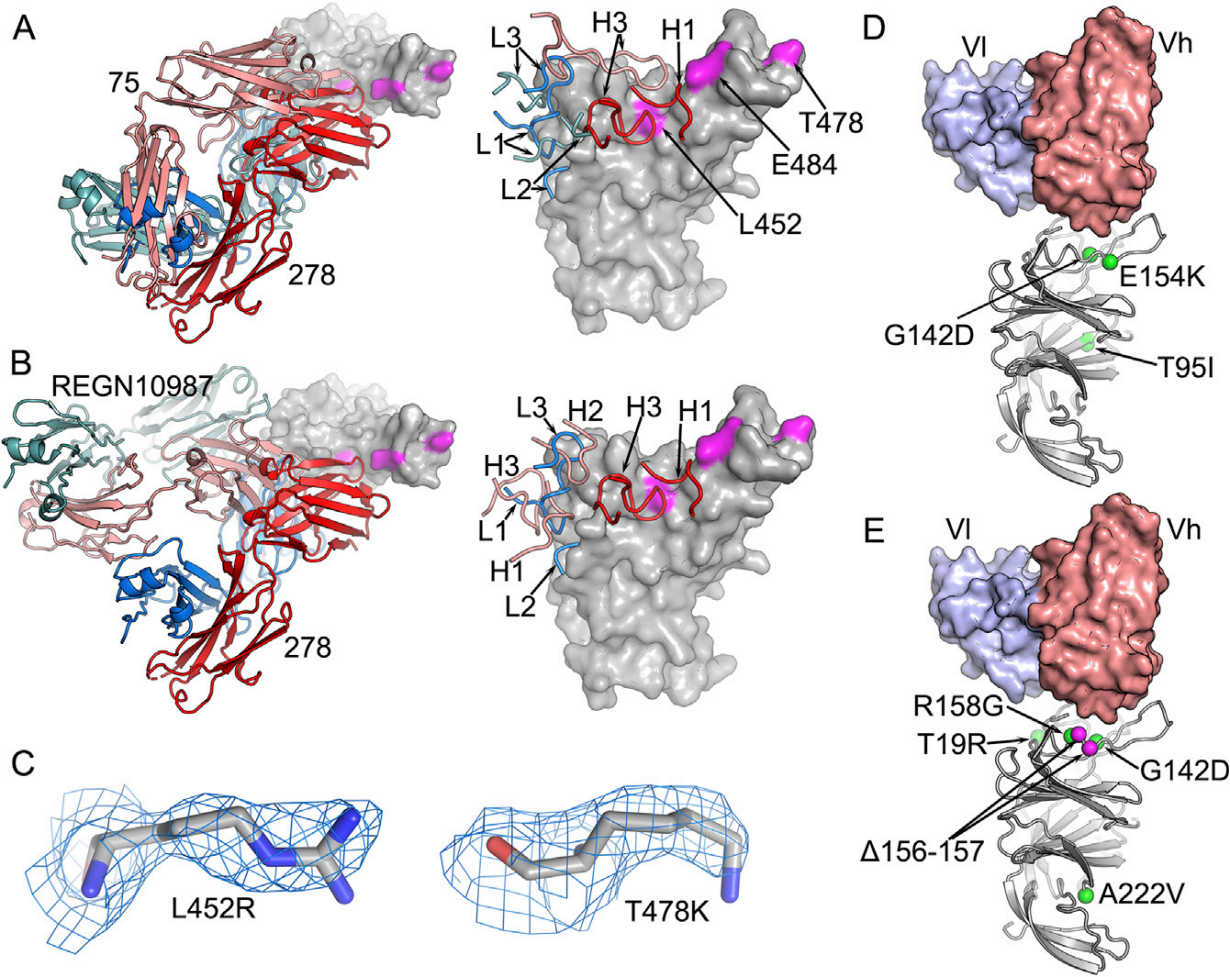
Structure features of SARS-CoV-2 mAbs and effects of B.1.617 mutations, related to Figure 4 (A) In the left panel, comparing the binding modes of fab 278 (red and blue) and Fab 75 (salmon and teal), and the right panel showing the CDR loops of the two Fabs involved in contacts with the RBD. The mutation sites, L452, T478 and E484, of B.1.617 variants are highlighted in magenta. (B) The left panel comparing the binding mode of fab 278 (red and blue) with that of REGN-10987 (salmon and teal, PDB ID 6XDG), and the right panel showing the CDR loops of the two Fabs involved in contacts with the RBD. (C) Electron density maps contoured at 1.0 σ showing the density for R452 in the L452R-RBD/75-253 complex (left), and K478 in the T478K-RBD/45-253 complex. (D), (E) Positions of the mutation sites in the NTD of the B.1.617.1 (D) and B.1.617.2 (E) spike relative to the bound antibody 159 (PDB ID 7NDC). The VhVl domains of mAb 159 are shown as surfaces and the NTD as gray ribbons with mutation and deletion sites marked with green and magenta spheres, respectively.

We performed three exemplar structural analyses to test our understanding of the physicochemical basis of antibody escape. First we determined the crystal structure (at 2.3-Å resolution) of Fab 278 in complex with Victoria RBD and Fab 222 (STAR Methods; Figures 4A–4D; Table S2). Neutralization and binding of mAb 278 are affected for B.1.617.1 and B.1.617.2, and we inferred that the mutation of RBD residue 452 was responsible. The structure confirms that neither RBD residue 478 nor 484 contact the antibody and that binding abrogation is mediated by direct contact between the 16-residue-long heavy chain (HC) complementarity determining region (CDR) 3 and RBD residue 452, which could not accommodate the major increase in side-chain size in going from leucine to arginine in the variant viruses (Figure 4C). Although REGN10987 and mAb 75 bind at a similar site as mAb 278, and all three antibodies overlap the ACE2 binding site (Baum et al., 2020; Dejnirattisai et al., 2021a), the engagement is sufficiently different that neither REGN10987 nor mAb 75 directly contact RBD residue 452 (Figure S2). REGN10987 is effective against B.1.617.1 and B.1.617.2, whereas mAb 75 is a weak binder. In fact, of the 13 different Fab complexes for which we have structures, only mAb 278 makes strong contacts with RBD residue 452; however, in addition, mAb 384 makes weak contacts with RBD residue 452 (Figures 4E and 4F) but more important contacts with residue 484. However, McCallum et al. (2021) report several RBD residue 452-interacting antibodies, and in our set of 20 potent neutralizers, we inferred interaction from the neutralization and binding data for three further mAbs (58, 170, and 281) for which we do not have structures, but competition mapping positioning is consistent with contact (Figure 3B; Dejnirattisai et al., 2021a), suggesting that such antibodies are not uncommon in responses to infection with Victoria-like viruses.

We determined the crystal structure of a ternary complex of RBD-L452R with Fabs 253 and 75 (STAR Methods; Figure 4G; Table S1). The RBD 452 mutation had no effect on neutralization or binding of mAb 253, and the structure confirms that the RBD L452R mutation introduces no significant change in the RBD structure and that residue 452 does not directly contact Fab 253 (Figure 4G). The third crystal structure is closely related; it is a ternary complex of Fab 253 with RBD-T478K and Fab 45 (Figure 4H; Table S2). Fab 253 is the only antibody whose binding is perturbed significantly by the mutation at RBD residue 478 (the closely related mAb 55 shows a similar but reduced effect), and the mutation to lysine actually increases the neutralization titer by approximately one log. Perhaps surprisingly, this is the only example we have come across of a marked increase in binding to a variant virus, and the structure confirms that this effect is due to direct interaction with RBD residue 478, with the lysine side chain in the variant RBD folding away behind the CDR1 loop of the light chain (LC) (Figures 4I and 4J). In addition, comparison of the overall mode of engagement of mAb 253 between the two differently mutated RBDs reveals that the 478 mutation induces a modest change in the pose of the antibody (Figure 4I). It is perhaps surprising that the threonine-to-lysine mutation of RBD residue 478, which represents a marked change in size and charge, has no deleterious effect on the binding of any of our set of potent mAbs. This suggests that antibody responses against Victoria-like viruses do not include a significant number of potent neutralizing antibodies that bind in this region, perhaps because this residue is toward the back of the left shoulder, facing away from the area where ACE2 attaches. Nevertheless, this residue is extremely exposed, and it is possible that responses in people infected by B.1.351 may produce a significant number of antibodies that interact with mutated RBD residue 484, some of which are likely to be sensitive to the mutation of RBD residue 478, perhaps contributing to the considerable antigenic distance between B.1.351 and B.1.617.2.

Finally, by reference to structures we have determined previously, we can confirm that antibody 316, which only loses neutralization of B.1.617.1, contacts the B.1.617.1-specific mutation E484 but not RBD residues L452 or T478 (Figures 4K and 4L).

### Neutralization of B.1.617.1 by convalescent serum

Deposited B.1.617.1 sequences are highly variable (COVID-19 Genomics UK (COG-UK) Consortium, 2020), so we constructed pseudoviruses containing three different B.1.617.1 S sequences. Compared with the Wuhan sequence, all share L452R and E484Q in the RBD together with D614G and P681R, which are the only substitutions in B.1.617-C; B.1.617-A has, in addition, E154K in the NTD plus E1072K and V1176F in S2; B.1.617-B contains T95I, G142D, and E154K in the NTD and Q1071H in S2 (Figure 1B).

We collected 4- to 9-week convalescent plasma from individuals infected during the first wave of infection in the United Kingdom before June 2020, plasma from individuals infected with B.1.1.7 in the United Kingdom (n = 18 confirmed by sequence or S gene knockout in diagnostic PCR), serum from cases of P.1 (n = 17 sequence confirmed) collected in Brazil, and serum from cases of B.1.351 collected from the United Kingdom and South Africa (n = 14; sequence confirmed, n = 12; isolated contacts of sequence-confirmed cases who developed infection during quarantine, n = 2) (Dejnirattisai et al., 2021a, 2021b; Supasa et al., 2021; Zhou et al., 2021).

**Figure 5:**
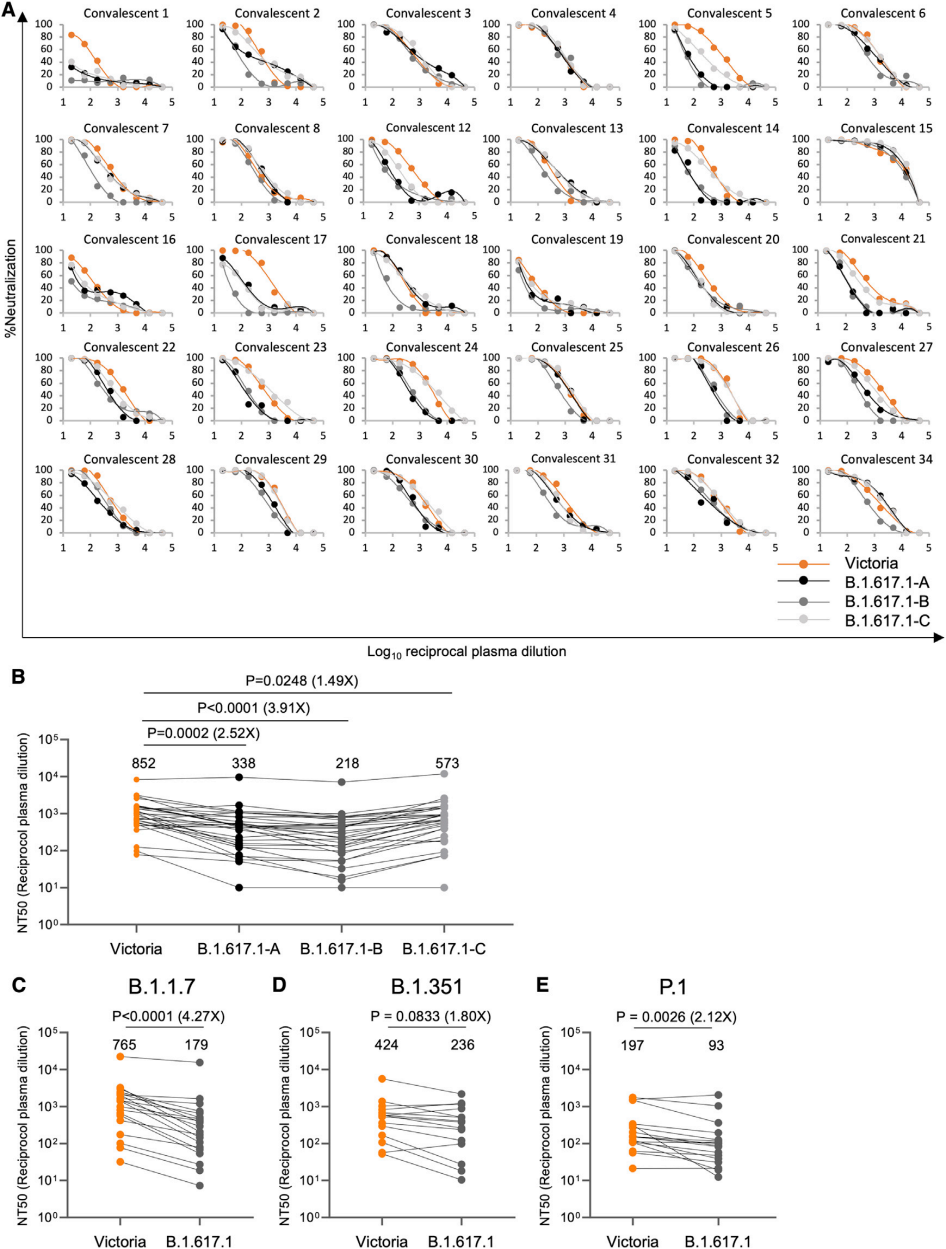
Neutralization of B.1.617.1 by convalescent serum (A) Neutralization of three (A, B, and C) B.1.617.1 pseudotyped lentiviruses by convalescent plasma (n = 34) collected from volunteers 4–9 weeks following SARS-CoV-2 infection; all samples were collected before June 2020 and therefore represent infection before the emergence of B.1.1.7 in the United Kingdom. Comparison is made with neutralization curves for pseudovirus Victoria. (B) Comparison of FRNT50 titers for B.1.617-A, B.1.617-B, and B.1.617-C with Victoria; geometric mean titers are shown above each column. (C and D) Neutralization titers for Victoria and B.1.617-B pseudovirus using (C) B.1.1.7 convalescent serum, (D) B.1.351 convalescent serum, and (C) P.1 convalescent serum. Wilcoxon matched-pairs signed-rank test was used for the analysis, and two-tailed p values were calculated. For the data presented for B.1.1.7 in (B), the sample with extremely high titers was excluded from the statistical analysis.

Neutralization of B.1.617.1 pseudoviruses was compared with neutralization of Victoria (Caly et al., 2020) using the United Kingdom samples taken in early 2020 (Figures 5A and 5B; Table S3; Dejnirattisai et al., 2021a). Relative to Victoria, geometric mean neutralization titers were reduced 2.5-fold (p = 0.0002) for B.1.617-A, 3.9-fold (p < 0.0001) for B.1.617.1-B, and 1.5-fold (p = 0.0248) for B.1.617-C. Differences in neutralization of three different B.1.617.1 subtypes may be due to mutations occurring in the NTD: 0 in B.1.617.1-C, 1 in B.1.617.1-A, and 3 in B.1.617-B, which was the most difficult to neutralize. B.1.617-B was used for subsequent experiments.

**Figure S3:**
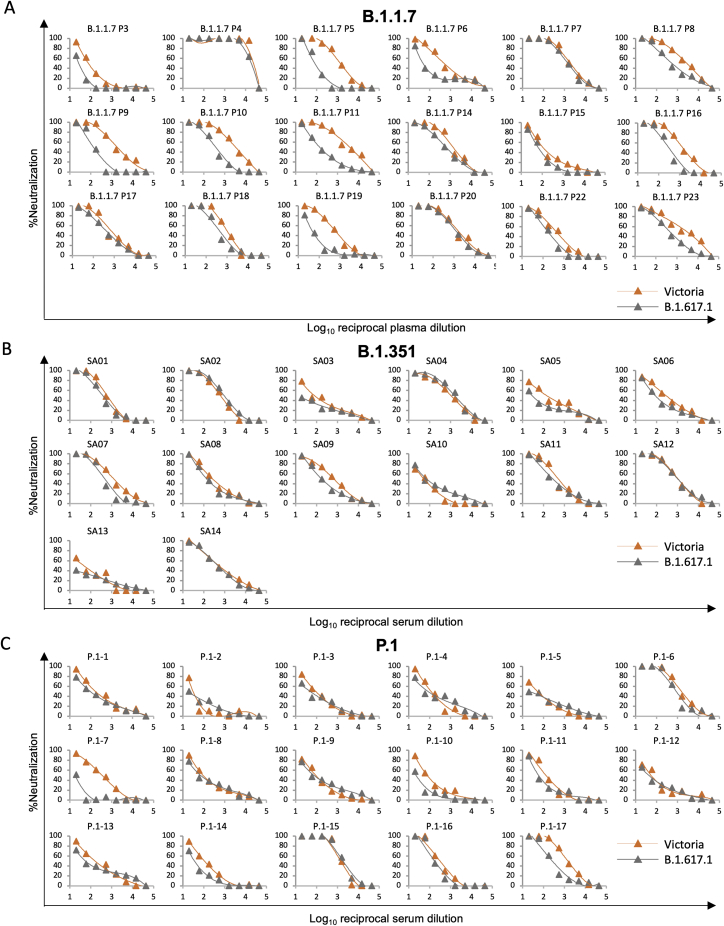
Neutralization curves against SARS-CoV-2 pseudotyped lentiviruses expressing full-length S of Victoria and B.1.617.1 strains by plasma from 18 individuals infected with B.1.1.7, serum from 14 individuals infected with B.1.351, and serum from 17 individuals infected with P.1, related to Figure 5 FRNT50 titers are given in Table S3.

Next we measured neutralization of B.1.617.1-B compared with Victoria by sera taken from individuals infected with B.1.1.7 (4.3-fold reduction [p < 0.0001]), B.1.351 (1.8-fold reduction [p = 0.0833]), and P.1 (2.1-fold reduction [p = 0.0026]), indicating that infection with these variant viruses provides substantial cross-protection against B.1.617.1, with no samples showing complete escape from neutralization (Figures 5C–5E; Figure S3).

### Neutralization of B.1.617.2 by convalescent serum

**Figure 6:**
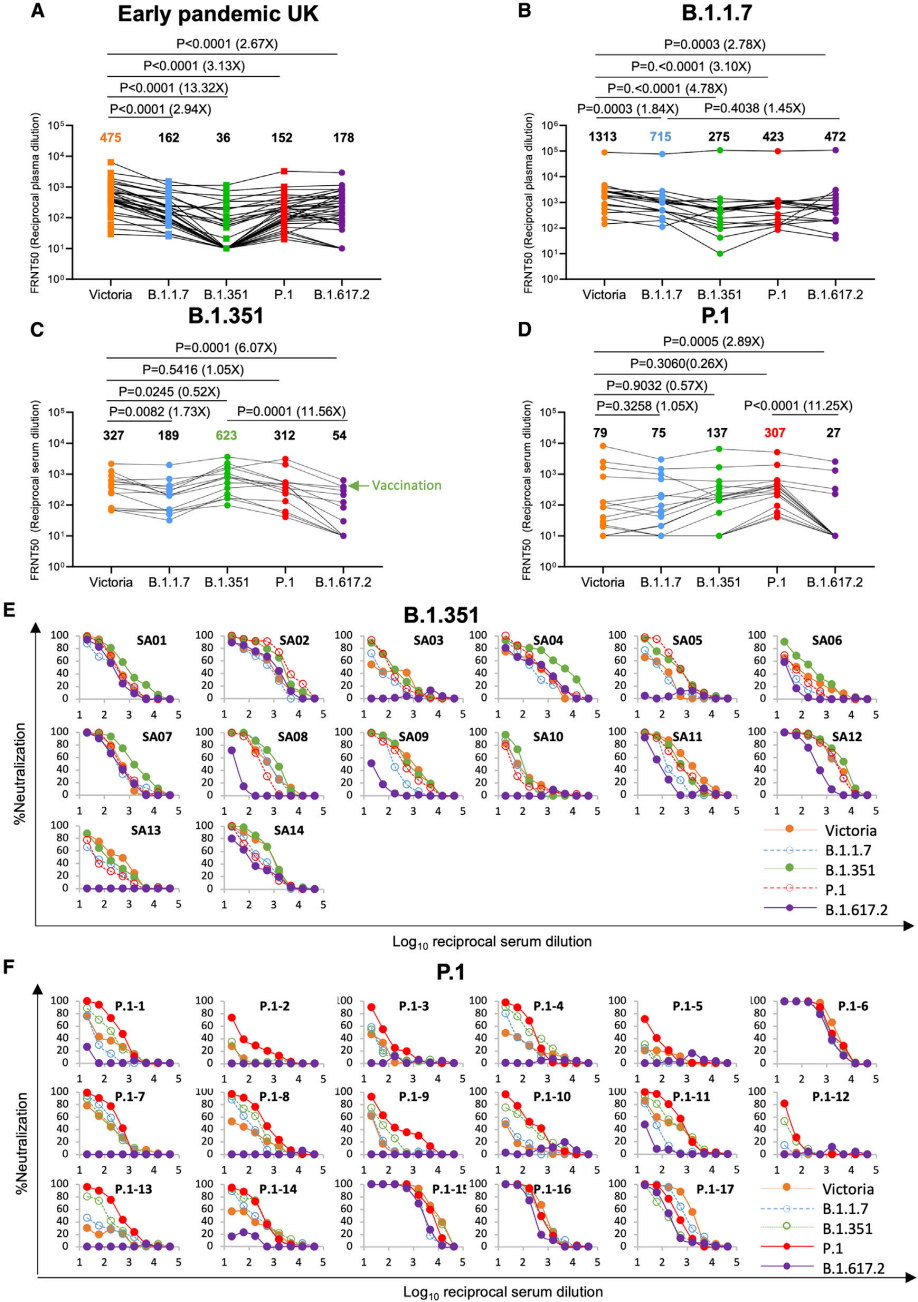
Neutralization of B.1.617.2 by convalescent plasma (A) Neutralization of B.1.617.2 live virus, measured by FRNT using the 34 convalescent samples described in Figure 5A; comparison is made with neutralization titers to Victoria, B.1.1.7, B.1.351, and P.1 (filled squares), reported previously in Supasa et al. (2021), Zhou et al. (2021), and Dejnirattisai et al. (2021b), and geometric mean titers are shown above each column. (B–D) Neutralization titers for Victoria, B.1.1.7, B.1.351, P.1, and B.1.617.2 using (B) B.1.1.7 convalescent plasma, (C) B.1.351 convalescent serum, and (D) P.1 convalescent serum. The green arrow in (C) represents serum from an individual who was infected with B.1.351 and subsequently received a vaccine. Wilcoxon matched-pairs signed-rank test was used for the analysis, and two-tailed p values were calculated. For the data presented for B.1.1.7 in (B), the sample with extremely high titers was excluded from the statistical analysis. (E and F) Neutralization curves for Victoria, B.1.1.7, B.1.351, P.1, and B.1.617.2 using convalescent serum from (E) B.1.351- and (F) P.1-infected individuals.

**Figure S4:**
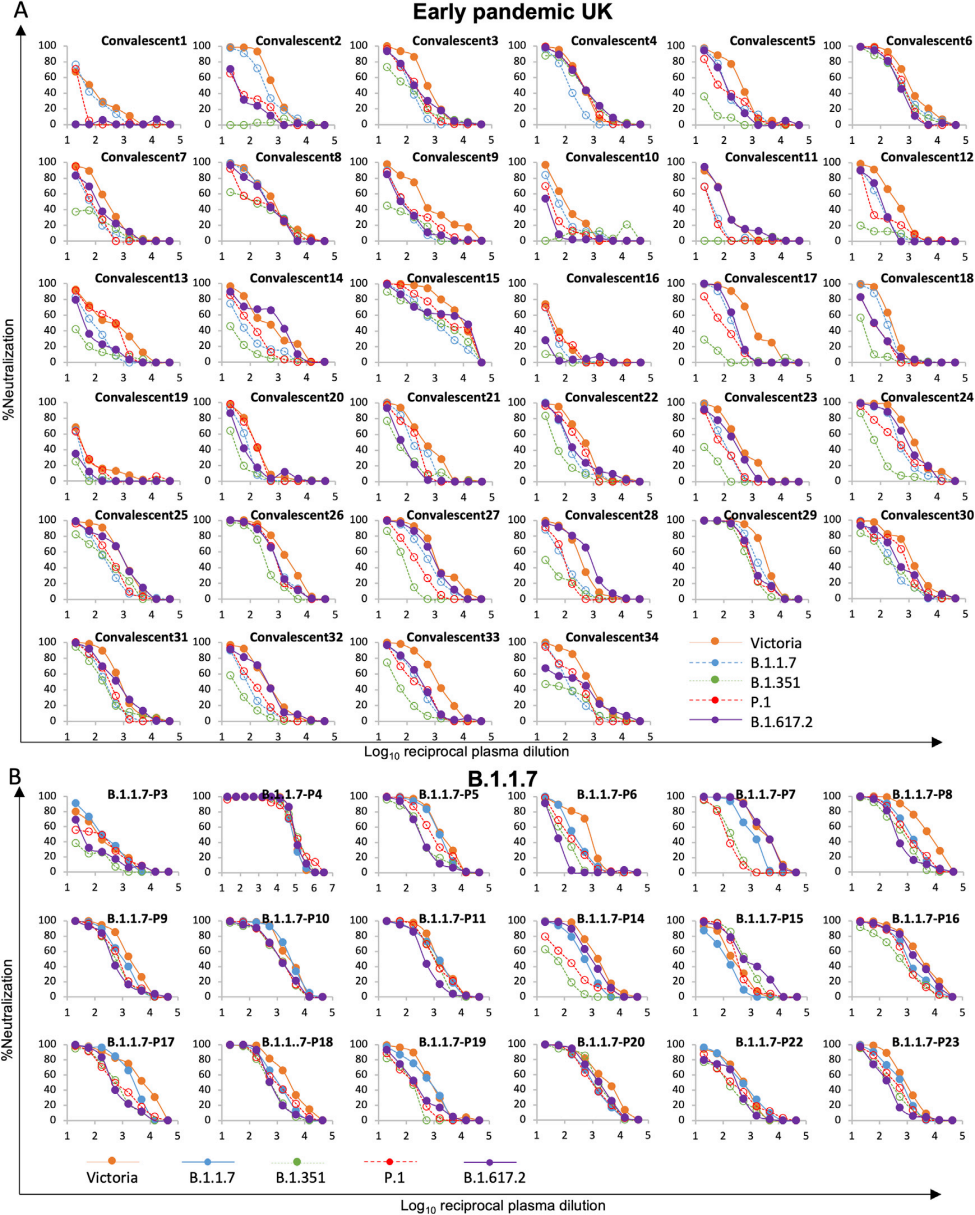
Neutralization curves against authentic SARS-CoV-2-Victoria, B.1.1.7, B.1.351, P.1. and B.1.617.2 strains by plasma from 34 individuals during the early pandemic in the United Kingdom and serum from 14 individuals infected with B.1.1.7, related to Figure 6 FRNT50 titers given in Table S3.

We measured neutralization of B.1.617.2 native virus on the same set of United Kingdom convalescent samples taken early during the pandemic (Figure 6A; Figure S4; Table S3). Compared with Victoria, geometric mean titers for B.1.617.2 were reduced 2.7-fold (p < 0.0001). Compared with Victoria, neutralization titers to B.1.617.2 were reduced for B.1.1.7 serum 2.8-fold (p = 0.0003), for B.1.351 serum 6.0-fold (p < 0.0001), and for P.1 serum 2.9-fold (p = 0.0005) (Figures 6B–6D; Table S3).

To get an idea of how people infected previously with B.1.1.7, B.1.351, and P.1 were protected from B.1.617.2, we compared the neutralization titers for B.1.617.2 with the neutralization of the homologous infecting lineage. For B.1.1.7 serum, neutralization of B.1.617.2 was reduced 1.5-fold (p = 0.4038) compared with B.1.1.7.; for B.1.351, serum neutralization was reduced 11.6-fold (p = 0.0001) compared with B.1.351; and for P.1, it was reduced 11.3-fold (p < 0.0001) compared with P.1 (Figures 6B–6D).

Serum from donors infected with B.1.1.7 appears to give good protection against all variants of concern, whereas protection from B.1.617.2 afforded by previous infection with B.1.351 and P.1 is much more compromised. Inspection of the neutralization curves using B.1.351 and P.1 serum (Figures 6E and 6F) shows that, in many cases, neutralization is lost almost completely to B.1.617.2, most profoundly for P.1, suggesting that individuals infected with B.1.351 and P.1 may be at risk of reinfection with B.1.617.2.

### Protection from B.1.617.1 and B.1.617.2 by vaccine serum

**Figure 7:**
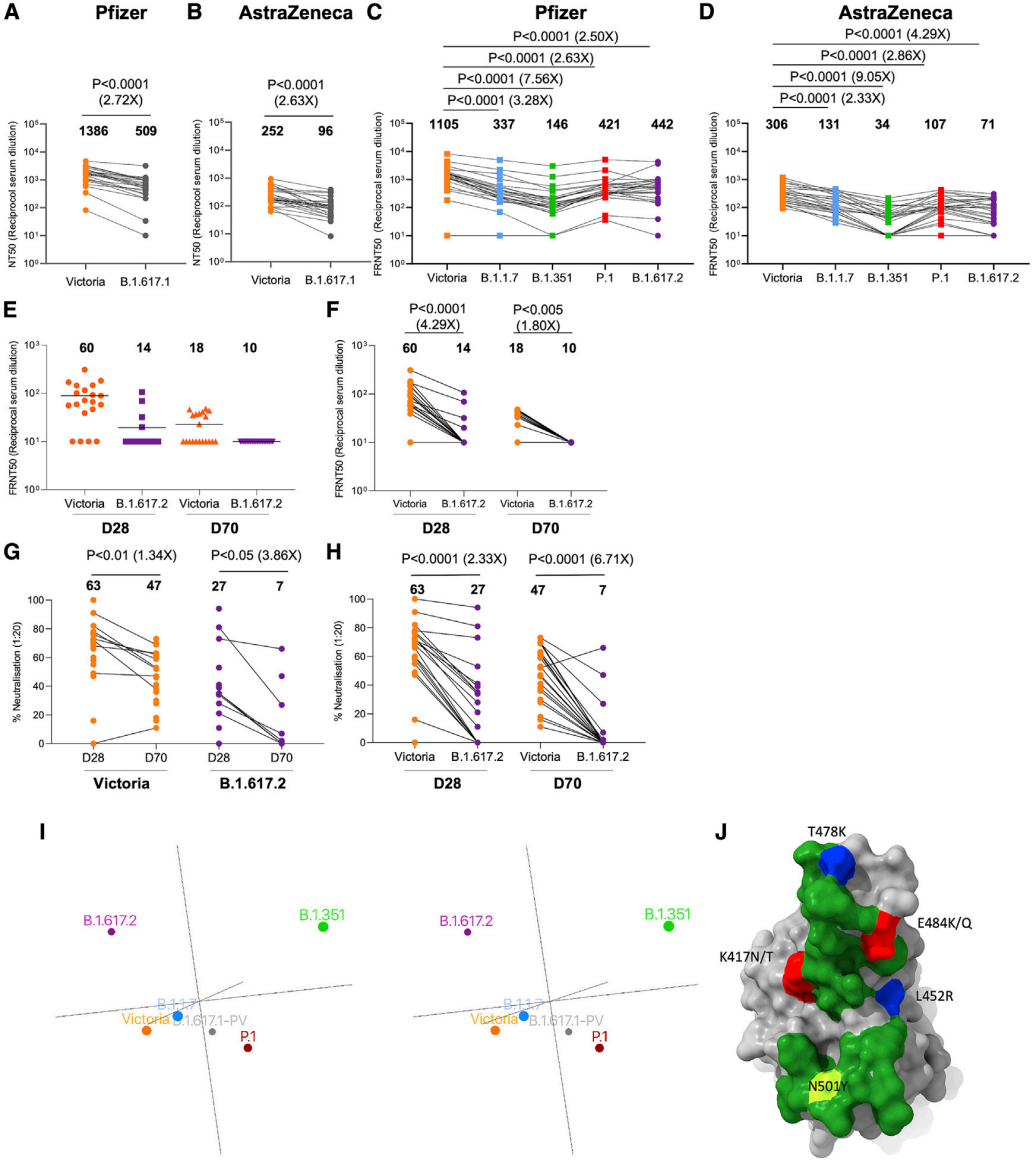
Neutralization by vaccine serum and mapping variants in antigenic space For the Pfizer vaccine, serum (n = 25) was taken 7–17 days following the second dose of the Pfizer-BioNTech vaccine. For the AstraZenca vaccine, serum was taken 14 or 28 days following the second dose of the Oxford-AstraZeneca vaccine (n = 25). (A) NT50 titers of Pfizer-BioNTech serum against B.1.617.1-B pseudovirus. (B) FRNT50 titers of Oxford-AstraZeneca serum against B.1.617.1-B pseudovirus. (C) FRNT50 titers of Pfizer-BioNTech serum against B.1.617.2 virus. (D) FRNT50 against of Oxford-AstraZeneca serum against B.1.617.2 virus. (A–D) Comparison is made with Victoria pseudo virus (A and B) or wild-type Victoria, B.1.1.7, B.1.351, and P.1 (filled squares), reported previously (Supasa et al., 2021; Zhou et al., 2021; Dejnirattisai et al., 2021b) (C and D). Subsequent panels analyze responses following a single dose of Pfizer vaccine. Serum (n = 20) was taken 28 or 70 days following the first dose of the Pfizer-BioNTech vaccine. (E and F) Comparison of FRNT50 titers for individual samples obtained 28 or 70 days after the first dose against Victoria or B.1.617.2. (G and H) Comparison of percent virus neutralization at serum dilution of 1:20 against SARS-CoV-2 Victoria and B.1.617.2 strains. Mean values are indicated above each column. (E–H) Mann-Whitney unpaired test was used for the analysis in (E) and (G). Wilcoxon matched-pairs signed rank test was used for the analysis in (F) and (H). (I) Map of variants in antigenic space. Wall-eyed stereo pair plots show output of principal-component analysis converting serum/virus strain pair neutralization capacities to antigenic space. Circle size denotes depth along the axis connecting the reader’s nose to the origin. See also Video S1. (J) Positions and charge effects of RBD mutations found in variants of concern. Shown is an incoming ACE2 view of the surface of the RBD, with the footprint of ACE2 shown in green and mutations occurring in variants, including B.1.1.7, P.1, P.1.351, B.1.617.1, and B.1.617.2, shown in a range of other colors.

**Figure S5:**
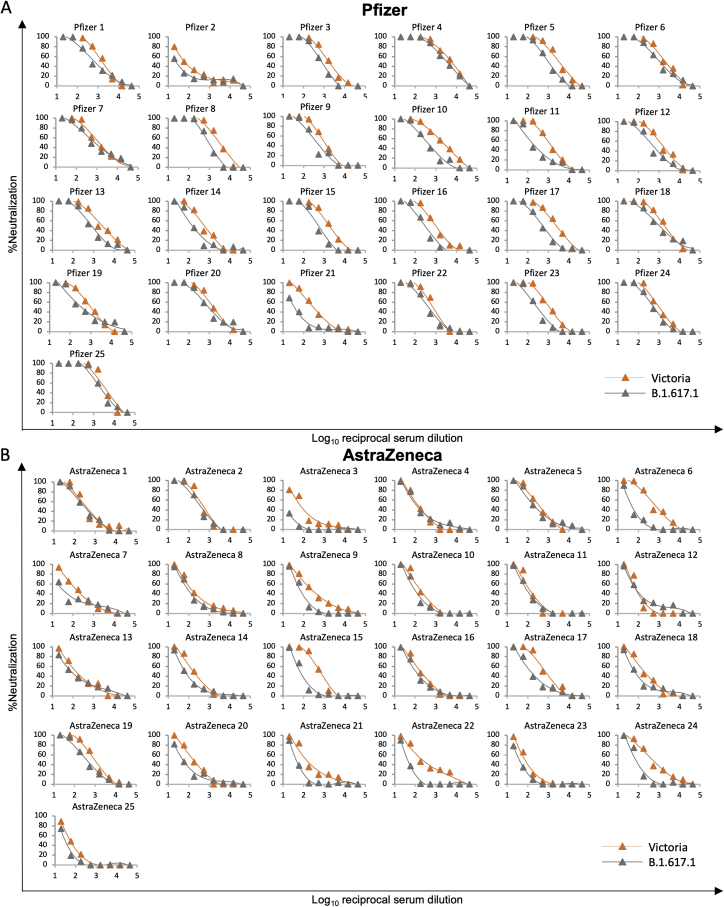
Neutralization curves against SARS-CoV-2 pseudotyped lentiviruses expressing full-length S of Victoria and B.1.617.1 strains by serum from 25 recipients of the Pfizer-BioNTech vaccine and Oxford-AstraZeneca vaccine, related to Figure 7 FRNT50 titers given in Table S4.

We tested neutralization of B.1.617.1 and B.1.617.2 using serum from individuals who had received 2 doses of the BNT162b2 Pfizer-BioNTech or ChAdOx1 nCoV-19 Oxford-AstraZeneca vaccine (Polack et al., 2020; Voysey et al., 2021). For Pfizer-BioNTech, serum was collected 4–14 days following the second dose of vaccine, administered 3 weeks after the first dose (n = 25). For the Oxford-AstraZeneca vaccine, serum was taken 14 or 28 days following the second dose, administered 8–14 weeks following the first dose (n = 25). Geometric mean neutralization titers against B.1.617.1 were reduced 2.7-fold (p < 0.0001) relative to the Victoria virus for the Pfizer-BioNTech vaccine serum (Figure 7A; Figure S5; Table S3) and 2.6-fold (p < 0.0001) for the Oxford-AstraZeneca vaccine (Figure 7B). For B.1.617.2, titers were reduced 2.5-fold (p < 0.0001) relative to the Victoria virus for the Pfizer-BioNTech vaccine serum (Figure 7C) and 4.3-fold (p < 0.0001) for the Oxford-AstraZeneca vaccine (Figure 7D). For B.1.617.2, reductions were comparable with those seen with B.1.1.7 and P.1 (Supasa et al., 2021; Dejnirattisai et al., 2021b), with only a small number of samples failing to reach FRNT50 titers at 1:20 serum dilution, in contrast to the results seen for neutralization of B.1.351 (Figure S4).

**Figure S6:**
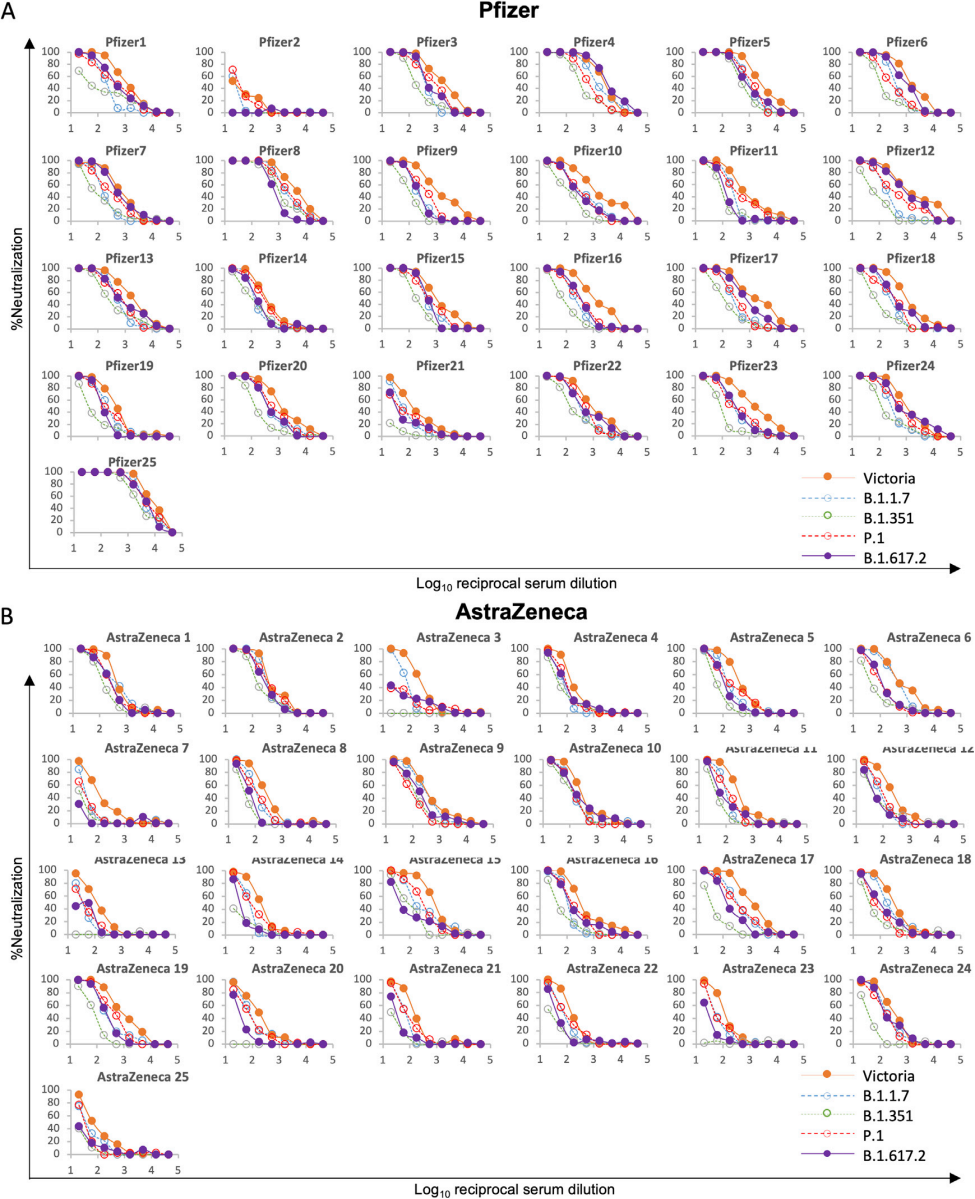
Neutralization curves against authentic SARS-CoV-2-Victoria, B.1.1.7, B.1.351, P.1, and B.1.617.2 strains by serum from 25 recipients of the Pfizer-BioNTech vaccine and Oxford-AstraZeneca vaccine, related to Figure 7 FRNT50 titers given in Table S4.

Finally, we performed neutralization assays using sera from volunteers 4 (n = 20) and 10 weeks (n = 20) after a single dose of the Pfizer-BioNTech vaccine. In the United Kingdom, for the Oxford-AstraZeneca and Pfizer-BioNTech vaccines, a dosing interval of 12 weeks is recommended to achieve higher vaccine coverage. Following one dose of the vaccine, neutralization of Victoria was observed in most individuals, with FRNT ≥ 50% in 16 of 20 individuals at 4 weeks and 9 of 20 individuals at 10 weeks. Titers against B.1.617.2 were lower, with FRNT ≥ 50% in 4 of 20 individuals at 4 weeks and 0 of 20 individuals at 10 weeks (Figures 7E and 7F). Peak neutralization titers at serum dilution of 1/20 were an average of 63% and 47% for Victoria and 27% and 7% for B.1.617.2 at 4 and 10 weeks, respectively, with many of the 10-week samples showing no evidence of neutralization of B.1.617.2 (Figures 7G and 7H).

### The antigenic landscape of the present major variants

To visualize and quantify the emerging antigenic landscape of SARS-CoV-2, we devised a method related to antigenic cartography (Smith et al., 2004; Fonville et al., 2014). We define “antigenic distance” by comparison of the log of dilution values for 50% neutralization for all available serum/virus strain pairs (Dejnirattisai et al., 2021a, 2021b; Supasa et al., 2021; Zhou et al., 2021). Three principal axes of variation, determined by single-value decomposition of this serum/virus strain matrix, were displayed to show the distribution of the strains in antigenic space. The result, using the somewhat incomplete set of data available from our studies, is shown in Figure 7I and Video S1. This provides a very simple view onto complex, sparse and noisy data and confirms the inferences made above: that the largest distance is between the B.1.351/P.1 lineages and B.1.617.2, whereas B.1.617.1 is significantly closer to B.1.351/P.1. Although B.1.351 is roughly orthogonal to B.1.617.2, P.1 is essentially opposite (anticorrelated with) B.1.617.2, reflecting the especially poor ability of P.1 serum to neutralize B.1.67.2. Note that B.1.1.7 and Victoria are reasonably central to the distribution.

Video S1. 3D view of the antigenic space plot for current variants, related to Figure 7I

## Discussion

The inevitable evolution of SARS-CoV-2 following its zoonotic transfer to humans in Wuhan in late 2019 prompted establishment of sequencing efforts such as COG-UK (COVID-19 Genomics UK (COG-UK) Consortium, 2020). SARS-CoV-2 genome surveillance in many parts of the world was slow to start, and there are many regions where surveillance is absent or completely underpowered compared with the scale of infections. It is likely that the true scale of the diversity in SARS-CoV-2 is underestimated and that further concerning variants are circulating and will continue to arise. Early in the pandemic, SARS-CoV-2 was under selective pressure to adapt to its new host, evade the innate immune system, efficiently bind to and infect target cells, and transmit to the next host. As the population develops immunity by natural infection or vaccination, pressure is mounting to select mutations that allow the virus to more effectively find an infectible host through increased transmissibility or evade the acquired immune response and cause reinfection.

Because the S protein is intimately involved in initiation of infection and is the target of neutralizing antibody responses, it is no surprise that it is evolving rapidly and that changes in S likely underpin some of the phenotypes expressed by variants of concern. S is a large protein of more than 1,200 amino acids, but a very small 25-amino-acid patch at its apex, mediating RBD/ACE2 interaction, is key. Mutations in and around the ACE2-interacting surface are found in all variants of concern (B.1.1.7, B.1.351, and P.1) as well as in the three B.1.617 sublineages (Figure 7H).

In this report, we measured the affinities for B.1.617.1 (L452R, E484Q), B.1.617.2 (L452R, T478K), and B.1.1.519 (T478K) RBDs for ACE2. The results show a very modest increase (less than 2-fold) in affinity for the variants. In contrast, we have previously measured the affinities of B.1.1.7 (N501Y), B.1.351 (N501Y, E484K, K417N) and P.1 (N501Y, E484K, K417T) RBDs for ACE2 and found more marked (7-, 19-, and 19-fold) increases in affinity, respectively, compared with Wuhan RBD, which may be driving the increased transmissibility of these strains. In line with our results, Zahradník et al., 2021 find that none of the three B.1.617 RBD mutations were selected by forced *in vitro* evolution to optimize ACE2 binding. It is therefore likely that the B.1.617.1 and B.1.617.2 RBD mutations were selected by different pressure. Nevertheless, the *in vitro* evolution experiments demonstrated that the increases in RBD/ACE2 affinity seen in today’s variants of concern are far from the limits that can be achieved, so in the future, more radical antigenic variation, which would render the virus unfit by reducing affinity for ACE2, might be rescued by employing these ACE2-binding enhancing mutations.

A hotspot for S sequence change is the NTD, with multiple changes occurring in tandem, consisting of amino acid substitutions together with small deletions and insertions. The NTD is the site of binding of a number of potently neutralizing antibodies whose mode of action is not yet fully understood because, unlike most potent anti-RBD antibodies, they do not block ACE2 interaction (Cerutti et al., 2021). B.1.617.2 has a highly mutated NTD (T19R, G142D, Δ156-157, R158G, A222V) that would be predicted to disrupt the so-called “super site” on the NTD mediating neutralization (Cerutti et al., 2021; Figure S2), and, in line with this, the neutralizing activity of mAb 159, which binds to the mutated area, is lost completely on B.1.617.2. B.1.617.1 sequences are quite variable, and here we examined three different isolates with 0, 1, or 3 mutations in the NTD, with the version containing 3 mutations being the most resistant to neutralization by convalescent plasma (Figure 1B; Figure S2).

Mutations L452R and E484Q knocked out activity of several potently neutralizing antibodies that bind to the RBD, but T478K, despite being relatively close in space to other key residues, such as 484, did not appear to have a negative effect on any of the panel of potent neutralizers. We solved the structure of a complex of the RBD with mAb 278 and confirm that this antibody contacts L452 (Figure 4A), explaining its loss of activity on B.1.617.1 and B.1.617.2. Furthermore, structures of other antibodies, such as 384, demonstrate reliance on contacts with L452 and E484, with the contacts with E484 probably dominant to L452 (Figure 4L). On the other hand, the structure of mAb 316 shows contact with E484 but no contact with L452 (Figure 4J). The light chain of mAb 253 contacts RBD residue T478, and the change at 478 enhances the binding/neutralization of B.1.617.2 (Figures 4G and 4H).

Because of the loss of activity of some potent neutralizing mAbs, we expected to see a reduction in neutralization of B.1.617.1 and B.1.617.2 by convalescent and vaccine sera. For B.1.617.1, we saw reductions of 3.9-fold for convalescent plasma, 2.7-fold for the Pfizer-BioNTech vaccine, and 2.6-fold for the Oxford-AstraZeneca vaccine; for B.1.617.2, reductions were 2.7-, 2.5-, and 4.3-fold, respectively. Reductions were comparable in scale with those seen with B.1.1.7 and P.1, with no evidence of widespread escape from neutralization, in contrast to that seen with B.1.351. It would seem likely from these results that the current RNA and viral vector vaccines will provide protection against the B.1.617 lineage, although an increase in breakthrough infections may occur as a result of the reduced neutralizing capacity of sera. Given the apparent high transmissibility of the variants, immunization of those at highest risk (older adults and those with co-morbidities) globally with at least one dose of the current generation of vaccines is urgently needed. It is known that the proportion of the population with strong neutralizing activity increases with a second dose (Folegatti et al., 2020), and we find that, following a single dose of the Pfizer-BioNTech vaccine, neutralization of B.1.617.2 is limited. Administration of two doses for those at greatest risk will therefore be needed to prevent infection. Infection with B.1.1.7 seems to provide reasonable cross-protection against all variants of concern, which means that B.1.1.7 might be a candidate for new variant vaccines to provide the broadest protection.

Of more concern was neutralization of B.1.617.2 by sera from people infected previously with B.1.351 and P.1, with 4 of 14 and 10 of 17 showing complete absence of neutralization of B.1.617.2, respectively. Although, in some cases, neutralization was knocked out for B.1.617.2, some sera showed almost no change in neutralization between B.1.315 or P.1 and the Victoria strain; determining at an epitope level how sera from these individuals differentially recognize variant viruses will be interesting. These results suggest that there is a risk of reinfection with B.1.617.2 in individuals infected previously by variants B.1.351 and P.1.

An explanation for the disparity in neutralization of B.1.617.2 by B.1.351 and P.1 serum may be that the differences between the two viruses are additive. Thus, there are three RBD amino acid substitutions in B.1.351 and P.1 compared with Wuhan RBD but five compared with B.1.351 and P.1 (the amino acid before the number represents the B.1.617.2 RBD sequence: K417N/T, R452L, K478T, N501Y, E484K). In addition, there are multiple differences in the NTDs, meaning that many antibodies generated by B.1.351 or P.1 infection will likely be ineffective against B.1.617.2. In B.1.617.1, there are 4 changes relative to B.1.351 and P.1 (K417N/T, L452R, N501Y, and Q484K), and it may be that lessening of the charge difference in RBD residue Q484K versus E484K and less pronounced differences in the NTDs make B.1.617.1 less resistant to neutralization by B.1.351 and P.1 serum. However as more variants emerge and robust serological data such as those presented here are obtained, it becomes essential to visualize and quantify the antigenic landscape of SARS-CoV-2 rather than rely on such increasingly complicated, narrative explanations. We present such a method (Figure 7J; Video S1), related to those called antigenic cartography. We define a multidimensional antigenic space representing “antigenic distances” within the sero-complex and show that, even using the incomplete data available, projecting the principal components into to a lower-dimensional space allows visualization of the antigenic relationships between the different lineages, confirming the qualitative assessment that the largest distance is between B.1.617.2 and the B.1.351/P.1 lineages (with P.1 being essentially anti-correlated with B.1.617.2), whereas B.1.617.1 is significantly closer to B.1.351/P.1. We suggest that the virus closest to the centroid of the distribution of antigenic differences might be a natural candidate for a vaccine antigen able to produce the most effective responses against all currently identified variants (in this case, it would be Wuhan or B.1.1.7). Useful extensions of the method might be to take account not only of antigenic distance but also of the nature and levels of the antibody responses against each virus. One striking outcome of this analysis is that clustering variants on the basis of antigenic distance gives completely different results from clustering by lineage, reflecting the major effect of a small number of mutations, which almost entirely switch the electrostatic properties by introducing basic residues around the edge of the ACE2 binding footprint on the RBD (Figure 7J).

The results showing reduced neutralization ability of serum derived from B.1.351 and P.1 individuals should drive consideration of policy decisions with new variant vaccines, when available, as it may indicate that the original “Wuhan” vaccine might be better than a B.1.351 vaccine for naive populations, even in areas where B.1.351 is the dominant variant. As SARS-CoV-2 continues to diverge antigenically, consideration is being given to booster vaccines to give further protection against viral variants such as B.1.351. How effective boosting will be to redirect the response toward the variants from the initial prime with Wuhan is to be determined. However, it is becoming more likely that more than one variant will be required to provide protection as the SARS-CoV-2 sero-complex continues to evolve; we suggest that one component will likely continue to include Wuhan-related strains or B.1.1.7 because, for now at least, they appear to be positioned more centrally in the sero-complex, able to provide protection against multiple virus variants.

Finally, we show a 1.34-fold reduction between 4 and 10 weeks in neutralization titers to Victoria in individuals given a single dose of the Pfizer-BioNTech vaccine and almost complete absence of neutralization of B.1.617.2 at 10 weeks. Previous studies have shown protection following a single dose of vaccine despite low or absent antibody responses, but recently, in the United Kingdom, some reduction in Pfizer-BioNTech effectiveness has been detected at 10 weeks (http://assets.publishing.service.gov.uk/government/uploads/system/uploads/attachment_data/file/988193/Vaccine_surveillance_report_-_week_20.pdf), presumably as a result of waning immunity, leading to the recommendation that the second vaccine dose interval should be reduced from 12 to 8 weeks in those over age 50 (https://www.gov.uk/government/news/jcvi-advice-to-mitigate-impact-of-b1-617-2-variant).

We report an in-depth study of antibody binding and neutralization of B.1.617.1 and B.1.617.2 viruses. Although there is a reduction in neutralization titers using convalescent or vaccine sera there is no evidence of widespread escape, suggesting that the current generation of vaccines will provide protection against the B.1.617 lineage, although reduced titers may lead to some breakthrough infections. However, there is concern that some unvaccinated individuals infected previously with B.1.351 and P.1 may be more at risk of reinfection with B.1.617.2. Further epidemiological data are needed to assess whether breakthrough infections following escape mutations are common and whether they will progress to severe disease and hospitalization. If this escape from the neutralizing capacity of vaccines continues with evolution of new variants in vaccinated populations and leads to a substantial reduction in effectiveness against hospitalization, there will be a significant effect on attempts to alter the course of the pandemic through immunization and an urgent need to revise immunogens.

### Limitations of the study

We compared pseudoviral neutralization data with live virus data, which is not ideal because some pseudoviral constructs did not recapitulate neutralization live-virus assays for mAbs, and we believe that live virus assays are preferable when available.

The *in vitro* neutralization assays described here are performed in the absence of complement or Fc receptor-bearing cells, which can mediate antibody-dependent cell-mediated cytotoxicity, meaning they may underestimate the protection of immune serum. Assays do not measure the T cell response, which may contribute to protection from severe disease and appear to be less disrupted by the changes in the variants of concern (Skelly et al., 2021). In the next few weeks, through careful studies in the United Kingdom, it will become clear how effective vaccines are at preventing B.1.617 infection and transmission and, crucially, progression to severe disease.

Further investigation of the antibody response in individuals infected with B.1.351 and P.1 is warranted to understand the complex cross-protective responses between different sera and variants; it will be particularly interesting to see how much the epitopes of neutralizing antibodies are skewed by infection with B.1.351 and P.1. Finally, the mechanism of neutralization of antibodies binding to the NTD and dissection of the role of antibodies to the NTD in neutralization are worthy of further investigation.

## STAR★Methods

### Key resources table

**Table:** 

REAGENT or RESOURCE	SOURCE	IDENTIFIER
**Antibodies**		

Fab	Dejnirattisai et al., 2021a	N/A
IgG	Dejnirattisai et al., 2021a	N/A
Human anti-NP (mAb 206)	Dejnirattisai et al., 2021a	N/A
Regeneron mAbs	AstraZeneca	Cat#REGN10933, and REGN10987
AstraZeneca mAbs	AstraZeneca	Cat#AZD1061, AZD8895
Vir mAbs	Adagio	Cat#S309
Lilly mAbs	Adagio	Cat#Ly-CoV555, and Cat#Ly-CoV16
Adagio mAbs	Adagio	Cat#ADG10, Cat#ADG20, and Cat#ADG30
Anti-Human IgG (Fc specific)-Peroxidase	Sigma	Cat#A0170
Polyclonal Goat Anti- human ACE2	R&D	Cat#AF933
Polyclonal Rabbit Anti-Goat Immunoglobulins/FITC	DAKO	Cat#F0250

**Bacterial and virus strains**		

SARS-CoV-2 (Australia/VIC01/2020)	Caly et al., 2020	N/A
SARS-CoV-2/B.1.1.7	Public Health England	N/A
SARS-CoV-2/B.1.351	Public Health England	N/A
SARS-CoV-2/P.1	This paper	N/A
DH5α bacteria	*In Vitro*gen	Cat#18263012

**Biological samples**		

Serum from Pfizer-vaccinated individuals	University of Oxford	N/A
Serum from AstraZeneca-Oxford-vaccinated individuals	University of Oxford	N/A
Plasma from SARS-CoV-2 patients	John Radcliffe Hospital in Oxford UK	N/A

**Chemicals, peptides, and recombinant proteins**		

His-tagged SARS-CoV-2 RBD	Dejnirattisai et al., 2021a	N/A
His-tagged SARS-CoV-2 RBD L452R, E484Q, T478K	This paper	N/A
His-tagged SARS-CoV-2 RBD L452R E484Q, L452R T478K	This paper	N/A
His-tagged human ACE2	This paper	N/A
Human ACE2-hIgG1Fc	This paper	N/A
Phosphate buffered saline tablets	Sigma-Aldrich	Cat#P4417
Dulbecco’s Modified Eagle Medium, high glucose	Sigma-Aldrich	Cat#D5796
Dulbecco’s Modified Eagle Medium, low glucose	Sigma-Aldrich	Cat#D6046
FreeStyle 293 Expression Medium	GIBCO	Cat#12338018
L-Glutamine–Penicillin–Streptomycin solution	Sigma-Aldrich	Cat#G1146
GlutaMAX Supplement	GIBCO	Cat#35050061
UltraDOMA PF Protein-free Medium	Lonza	Cat#12-727F
Opti-MEM	GIBCO	Cat#11058021
Fetal Bovine Serum	GIBCO	Cat#12676029
Polyethylenimine, branched	Sigma-Aldrich	Cat#408727
Carboxymethyl cellulose	Sigma	Cat#C4888
Strep-Tactin®XT	IBA Lifesciences	Cat#2-1206-025
HEPES	Melford	Cat#34587-39108
Sodium Chloride	Honeywell	Cat#SZBF3340H
LB broth	Fisher Scientific UK	Cat#51577-51656
Mem Neaa (100X)	GIBCO	Cat#2203945
Trypsin-EDTA	GIBCO	Cat#2259288
TrypLE Express Enzyme	GIBCO	Cat#12604013
L-Glutamine 200 mM (100X)	GIBCO	Cat#2036885
SYPROorange (5000X in DMSO)	Thermo	Cat#S6651
Isopropyl β-d-1-thiogalactopyranoside	Meridian Bioscience	Cat#BIO-37036
Kanamycin	Melford	Cat#K22000
Lysozyme	Sigma-Aldrich	Cat#L6876
Tris-base	Melford	Cat#T60040
Imidazole	Sigma-Aldrich	Cat#56750
Triton X-100	Sigma-Aldrich	Cat#8787
Turbonuclease	Sigma-Aldrich	Cat#T4330
RNase A	QIAGEN	Cat#158922
NaCl	Sigma-Aldrich	Cat#S9888
MgSO4	Sigma-Aldrich	Cat#746452
Na2HPO4	Melford	Cat#S23100
NaH2PO4	Melford	Cat#S23185

**Critical commercial assays**		

Bright-Glo Luciferase Assay System	Promega	Cat#E2620
HIV Type 1 p24 Antigen ELISA 2.0	ZeptoMetrix	Cat#0801002

**Deposited data**		

Crystal structures of SARS-CoV-2 RBD/Fab complexes	This paper	PDBs:7OR9,7ORA,7ORB

**Experimental models: Cell lines**		

HEK293S GnTI- cells	ATCC	Cat#CRL-3022
HEK293 cells	ATCC	Cat#CRL-3216
Expi293F Cells	GIBCO,	Cat#A14527
HEK293T/17 cells	ATCC	Cat#CRL-11268
HEK293T cells	ATCC	Cat#CRL-11268
Hamster: ExpiCHO cells	Thermo Fisher	Cat#A29133
Vero cells	ATCC	Cat#CCL-81

**Recombinant DNA**		

Vector: pHLsec	Aricescu et al., 2006	N/A
Vector: pNEO	Aricescu et al., 2006	N/A
Vector: p8.91	di Genova et al., 2020	Nigel Temperton
Vector: pCSFLW	di Genova et al., 2020	Nigel Temperton
Vector: pcDNA-SARS-CoV-2 spike	di Genova et al., 2020	Nigel Temperton
Vector: pcDNA-SARS-CoV-2 spike of Victoria strain	This paper	N/A
Vector: pcDNA-SARS-CoV-2 spike of B.1.617.1A strain	This paper	N/A
Vector: pcDNA-SARS-CoV-2 spike of B.1.617.1B strain	This paper	N/A
Vector: pcDNA-SARS-CoV-2 spike of B.1.617.1C strain	This paper	N/A
Vector: pcDNA-SARS-CoV-2 spike of B.1.617.2 strain	This paper	N/A
Vector: pcDNA-SARS-CoV-2 spike of B.1.1.519	This paper	N/A
Vector: pcDNA-SARS-CoV-2 spike of B.1.429	This paper	N/A
Vector: pCMV-VSV-G	Stewart et al., 2003	Addgene plasmid # 8454
pHR-SIN-ACE2	Alain Townsend	N/A
Vector: pOPING-ET	Nettleship et al., 2008	N/A
human ACE2 cDNA	Sourcebiosciences	Cat#5297380
Vector: human IgG1 heavy chain	German Cancer Research Center, Heidelberg, Germany (H. Wardemann	N/A
Vector: human lambda light chain	German Cancer Research Center, Heidelberg, Germany (H. Wardemann	N/A
Vector: human kappa light chain	German Cancer Research Center, Heidelberg, Germany (H. Wardemann	N/A
Vector: Human Fab	Univeristy of Oxford	N/A
Vector: Human scFv	University of Oxford, NDM (G. Screaton)	N/A

**Software and algorithms**		

COOT	Emsley and Cowtan, 2004	https://www2.mrc-lmb.cam.ac.uk/personal/pemsley/coot/
Xia2-dials	Winter et al., 2018	https://xia2.github.io/parameters.html
PHENIX	Liebschner et al., 2019	https://phenix-online.org/
PyMOL	Schrodinger	https://pymol.org/2/
Data Acquisition Software 11.1.0.11	Fortebio	https://www.sartorius.com/en/products/protein-analysis/octet-systems-software
Data Analysis Software HT 11.1.0.25	Fortebio	https://www.sartorius.com/en/products/protein-analysis/octet-systems-software
Prism 8.0	GraphPad	https://www.graphpad.com/scientific-software/prism/
IBM SPSS Software 26	IBM	https://www.ibm.com/us-en/?ar=1
Mabscape	This paper	https://github.com/helenginn/mabscape; https://snapcraft.io/mabscape

**Other**		

X-ray data were collected at beamline I03, Diamond Light Source, under proposal ib27009 for COVID-19 rapid access	This paper	https://www.diamond.ac.uk/covid-19/for-scientists/rapid-access.html
TALON Superflow Metal Affinity Resin	Clontech	Cat#635668
HiLoad 16/600 Superdex 200 pg	Cytiva	Cat#28-9893-35
Superdex 200 increase 10/300 GL column	Cytiva	Cat#28990944
HisTrap HP 5-ml column	Cytiva	Cat#17524802
HiTrap Heparin HT 5-ml column	Cytiva	Cat#17040703
Amine Reactive Second-Generation (AR2G) Biosensors	Fortebio	Cat#18-5092
Octet RED96e	Fortebio	https://www.sartorius.com/en/products/protein-analysis/octet-label-free-detection-systems
Buffer exchange system “QuixStand”	GE Healthcare	Cat#56-4107-78
Cartesian dispensing system	Genomic solutions	Cat#MIC4000
Hydra-96	Robbins Scientific	Cat#Hydra-96
96-well crystallization plate	Greiner bio-one	Cat#E20113NN
Crystallization Imaging System	Formulatrix	Cat#RI-1000
Sonics vibra-cell vcx500 sonicator	VWR	Cat#432-0137

### Resource availability

#### Lead contact

Resources, reagents and further information requirement should be forwarded to and will be responded by the Lead Contact, David I Stuart (dave@strubi.ox.ac.uk).

#### Materials availability

Reagents generated in this study are available from the Lead Contact with a completed Materials Transfer Agreement.

#### Data and code availability

The coordinates and structure factors of the crystallographic complexes are available from the PDB with accession codes: 7OR9, 7ORA, 7ORB (see Table S1). Mabscape is available from https://github.com/helenginn/mabscape, https://snapcraft.io/mabscape. The data that support the findings of this study are available from the corresponding authors on request.

### Experimental model and subject details

#### Viral stocks

SARS-CoV-2/human/AUS/VIC01/2020 (Caly et al., 2020), SARS-CoV-2/B.1.1.7 and SARS-CoV-2/B.1.351 were provided by Public Health England, P.1 from a throat swab from Brazil were grown in Vero (ATCC CCL-81) cells. Cells were infected with the SARS-CoV-2 virus using an MOI of 0.0001. Virus containing supernatant was harvested at 80% CPE and spun at 3000 rpm at 4°C before storage at −80°C. Viral titers were determined by a focus-forming assay on Vero cells. Victoria passage 5, B.1.1.7 passage 2 and B.1.351 passage 4 stocks P.1 passage 1 stocks were sequenced to verify that they contained the expected spike protein sequence and no changes to the furin cleavage sites. The B.1.617.2 virus was kindly provided Wendy Barclay and Thushan De Silva contained the following mutations compared to the Wuhan sequence T19R, G142D, Δ156-157/R158G, A222V, L452R, T478K, D614G, P681R, D950N.

#### Bacterial strains and cell culture

Vero (ATCC CCL-81) cells were cultured at 37°C in Dulbecco’s Modified Eagle medium (DMEM) high glucose (Sigma-Aldrich) supplemented with 10% fetal bovine serum (FBS), 2 mM GlutaMAX (GIBCO, 35050061) and 100 U/ml of penicillin–streptomycin. Human mAbs were expressed in HEK293T cells cultured in UltraDOMA PF Protein-free Medium (Cat# 12-727F, LONZA) at 37°C with 5% CO_2_. *E.coli DH5α* bacteria were used for transformation of plasmids encoding wt and mutated RBD proteins. A single colony was picked and cultured in LB broth with 50 μg mL^-1^ Kanamycin at 37°C at 200 rpm in a shaker overnight. HEK293T (ATCC CRL-11268) cells were cultured in DMEM high glucose (Sigma-Aldrich) supplemented with 10% FBS, 1% 100X Mem Neaa (GIBCO) and 1% 100X L-Glutamine (GIBCO) at 37°C with 5% CO_2_. To express RBD, RBD variants and ACE2, HEK293T cells were cultured in DMEM high glucose (Sigma) supplemented with 2% FBS, 1% 100X Mem Neaa and 1% 100X L-Glutamine at 37°C for transfection.

#### Plasma from early pandemic and B.1.1.7 cases

Participants from the first wave of SARS-CoV2 in the UK and those sequence confirmed with B.1.1.7 lineage in December 2020 and February 2021 were recruited through three studies: Sepsis Immunomics [Oxford REC C, reference:19/SC/0296]), ISARIC/WHO Clinical Characterization Protocol for Severe Emerging Infections [Oxford REC C, reference 13/SC/0149] and the Gastro-intestinal illness in Oxford: COVID sub study [Sheffield REC, reference: 16/YH/0247]. Diagnosis was confirmed through reporting of symptoms consistent with COVID-19 and a test positive for SARS-CoV-2 using reverse transcriptase polymerase chain reaction (RT-PCR) from an upper respiratory tract (nose/throat) swab tested in accredited laboratories. A blood sample was taken following consent at least 14 days after symptom onset. Clinical information including severity of disease (mild, severe or critical infection according to recommendations from the World Health Organization) and times between symptom onset and sampling and age of participant was captured for all individuals at the time of sampling. Following heat inactivation of plasma/serum samples they were aliquoted so that no more than 3 freeze thaw cycles were performed for data generation.

#### Sera from B.1.351 and P.1infected cases

B.1.351 samples from UK infected cases was collected under the “Innate and adaptive immunity against SARS-CoV-2 in healthcare worker family and household members” protocol affiliated to the Gastro-intestinal illness in Oxford: COVID sub study discussed above and approved by the University of Oxford Central University Research Ethics Committee. All individuals had sequence confirmed B.1.351 infection or PCR-confirmed symptomatic disease occurring while in isolation and in direct contact with B.1.351 sequence-confirmed cases. Additional B.1.351 infected serum (sequence confirmed) was obtained from South Africa. At the time of swab collection patients signed an informed consent to consent for the collection of data and serial blood samples. The study was approved by the Human Research Ethics Committee of the University of the Witwatersrand (reference number 200313) and conducted in accordance with Good Clinical Practice guidelines. P.1 samples were provided by the International Reference Laboratory for Coronavirus at FIOCRUZ (WHO) as part of the national surveillance for coronavirus and had the approval of the FIOCRUZ ethical committee (CEP 4.128.241) to continuously receive and analyze samples of COVID-19 suspected cases for virological surveillance. Clinical samples were shared with Oxford University, UK under the MTA IOC FIOCRUZ 21-02.

#### Sera from Pfizer vaccinees

Pfizer vaccine serum was obtained from volunteers who had received either one or two doses of the BNT162b2 vaccine. Vaccinees were Health Care Workers, based at Oxford University Hospitals NHS Foundation Trust, not known to have prior infection with SARS-CoV-2 and were enrolled in the OPTIC Study as part of the Oxford Translational Gastrointestinal Unit GI Biobank Study 16/YH/0247 [research ethics committee (REC) at Yorkshire & The Humber – Sheffield]. The study was conducted according to the principles of the Declaration of Helsinki (2008) and the International Conference on Harmonization (ICH) Good Clinical Practice (GCP) guidelines. Written informed consent was obtained for all participants enrolled in the study. Two groups were studied after receiving COVID-19 mRNA Vaccine BNT162b2, 30 μg, administered intramuscularly after dilution (0.3 mL each). A “short dosing interval” group were sampled 7-17 days after receiving two doses of vaccine 18-28 days apart, and a “long dosing interval” group were sampled twice, approximately 28 days (range 25-35) and 70 days (range 48-93) after receiving a single dose of the vaccine. The mean age of vaccines was 37 years (range 22-66), 21 male and 35 female.

#### AstraZeneca-Oxford vaccine study procedures and sample processing

Full details of the randomized controlled trial of ChAdOx1 nCoV-19 (AZD1222), were previously published (Ramasamy et al., 2021; Folegatti et al., 2020). These studies were registered at ISRCTN (15281137 and 89951424) and ClinicalTrials.gov (NCT04324606 and NCT04400838). Written informed consent was obtained from all participants, and the trial is being done in accordance with the principles of the Declaration of Helsinki and Good Clinical Practice. The studies were sponsored by the University of Oxford (Oxford, UK) and approval obtained from a national ethics committee (South Central Berkshire Research Ethics Committee, reference 20/SC/0145 and 20/SC/0179) and a regulatory agency in the United Kingdom (the Medicines and Healthcare Products Regulatory Agency). An independent DSMB reviewed all interim safety reports. A copy of the protocols was included in previous publications (Ramasamy et al., 2021; Folegatti et al., 2020).

Data from vaccinated volunteers who received two vaccinations are included in this paper. Vaccine doses were either 5 × 10^10^ viral particles (standard dose; SD/SD cohort n = 21) or half dose as their first dose (low dose) and a standard dose as their second dose (LD/SD cohort n = 4). The interval between first and second dose was in the range of 8-14 weeks. Blood samples were collected and serum separated on the day of vaccination and on pre-specified days after vaccination e.g., 14 and 28 days after boost.

### Method details

#### Focus Reduction Neutralization Assay (FRNT)

The neutralization potential of Ab was measured using a Focus Reduction Neutralization Test (FRNT), where the reduction in the number of the infected foci is compared to a negative control well without antibody. Briefly, serially diluted Ab or plasma was mixed with SARS-CoV-2 strain Victoria or P.1 and incubated for 1 hr at 37°C. The mixtures were then transferred to 96-well, cell culture-treated, flat-bottom microplates containing confluent Vero cell monolayers in duplicate and incubated for a further 2 hr followed by the addition of 1.5% semi-solid carboxymethyl cellulose (CMC) overlay medium to each well to limit virus diffusion. A focus forming assay was then performed by staining Vero cells with human anti-NP mAb (mAb206) followed by peroxidase-conjugated goat anti-human IgG (A0170; Sigma). Finally, the foci (infected cells) approximately 100 per well in the absence of antibodies, were visualized by adding TrueBlue Peroxidase Substrate. Virus-infected cell foci were counted on the classic AID EliSpot reader using AID ELISpot software. The percentage of focus reduction was calculated and IC_50_ was determined using the probit program from the SPSS package.

#### Plasmid construction and pseudotyped lentiviral particles production

The constructs of pseudotyped lentivirus expressing SARS-CoV-2 S proteins are as previously described in Nie et al. (2020), with some modifications. Briefly, the gene sequences were designed to encode S protein of Victoria (S247R), B.1.617.1A (E154K, L452R, E484Q, D614G, P681R, E1072K and K1073R), B.1.617.1B (T95I, G142D, E154K, L452R, E484Q, D614G, P681R and Q1071H), B.1.617.1C (L452R, E484Q, D614G and P681R), B.1.617.2 (T19R, 156-158del, L452R, T478K, D614G, P681R and D950N), B.1.1.519 (T478K, D614G, P681H and T732A) or B.1.429 (S13I, W152C, L452R and D614G). A synthetic codon-optimized SARS-CoV-2 construct from Wuhan-Hu-1 (GenBank: MN908947) was used as the template and the constructs were cloned by PCR amplification of vector and inserts, followed by Gibson assembly. To generate the insert fragments, the overlapping primers for all individual variants were used separately to amplify, together with two primers of pcDNA3.1 vector (pcDNA3.1_BamHI_F and pcDNA3.1_Tag_S_EcoRI_R). The pcDNA3.1 vector was also amplified using pcDNA3.1_Tag_S_EcoRI_F and pcDNA3.1_BamHI_R primers. The primer pairs used in this study are shown in supplementary (Table S5). All constructs were verified by Sanger sequencing.

Production of pseudotyped lentiviral particles expressing SARS-CoV-2 S protein was carried out as described previously (di Genova et al., 2020), with some modifications. Briefly, HEK293T/17 cells (ATCC® CRL-11268) were co-transfected with three essential plasmids; plasmid (pCDNA 3.1) expressing SARS-CoV-2 S protein (Victoria or B.1.617.1 or B.1.1.519), lentiviral vector expressing firefly luciferase reporter protein (pCSFLW), and the second generation of lentiviral packaging plasmid (p8.91) expressing gag, pol and rev proteins at the ratio of 1:1.5:1 μg, respectively, in 200 μl opti-MEM (GIBCO). The DNA cocktails were then supplemented with the equal volume of opti-MEM containing 35 μL of 1 mg/mL polyethylenimine (Sigma-Aldrich). After 20 min incubation, the plasmid DNA-PEI complexes were then added into the T75 cm^2^ culture flask containing approximate 50% confluency of HEK293T/17 cells. The medium was changed twice, one hour prior to transfection and 18-24 h post transfection. The culture supernatant containing pseudotyped lentiviral particles were harvested at 72 h post-transfection by centrifugation and kept at −80°C. In each experiment, 90 ng/mL of HIV- gag protein was normalized using the RETROtek HIV-1 p24 Antigen-ELISA kit (Zeptometrix; Buffalo, NY), according to manufacturer’s instructions.

A similar strategy was used to produce lentiviral vector carrying human ACE2 (hACE2). However, the plasmid expressing SARS-CoV-2 S and luciferase reporter proteins were replaced by the Vesicular stomatitis virus G protein (pCMV-VSV-G) and vector expressing human ACE2 (pHR-SIN-ACE2). Both plasmids were kindly provided by Alain Townsend. The resulting lentiviral particles were transduced into HEK293T/17 cells to generate the stable expressing hACE2 receptor. The transduced cells were subjected to hACE2 staining and single cell sorting, clones with > 80% hACE2 positive cells were used as the target cells for pseudotyped based neutralization assays.

#### Pseudoviral neutralization assay

Pseudotyped lentiviral particles expressing SARS-CoV2 S protein (Victoria or B.1.617 or B.1.1.519) were incubated with serial dilutions of mAbs or plasma in white opaque 96-well plates at 37°C, 5% CO2 for 1 hr. The stable HEK293T/17 cells expressing human ACE2 were then added to the mixture at 1.5x10^4^ cells/well. Plates were spun at 500 RCF for 1 min and further incubated for another 48 hr. Culture supernatants were removed and 50 μL of 1:2 Bright-GloTM Luciferase assay system (Promega, USA) in 1X PBS (sigma) was added to each well. The reaction was incubated at room temperature for 5 mins and the firefly luciferase activity was measured using CLARIOstar® (BMG Labtech). The percentage of neutralization of mAbs or plasma samples toward pseudotyped lentiviruses was calculated relative to the control.

#### Cloning of ACE2 and RBD proteins

The constructs of ACE2, WT RBD, B.1.1.7, B.1.351 and P.1 mutant RBD are the same as previously described (Dejnirattisai et al., 2021, Zhou et al., 2021, Supasa et al., 2021). To clone RBD expression plasmids which has the same nucleotide optimization with the spike of pseudovirus (RBD-PV), the sRBD fragment were amplified from pcDNA 3.1- SARS-CoV-2 Spike plasmids using primers of PV-RBD. pNEO vector was digested by AgeI and KpnI and joined with RBD fragments by Gibson assembly.

To construct RBD L452R and T478K, primers of L452R and primers of T478K were used separately, together with two primers of pNEO vector to do PCR, with the plasmid of RBD-PV as the template. To construct RBD L452R T478K and RBD L452R E484Q, primers of T478K and RBD E484Q were used to pair with the primers of pNEO vector to do PCR, with the plasmid of RBD L452R as template. Two PCR fragments amplified for each mutation were purified by QIAquick Gel Extraction Kit (QIAGEN, Hilden, Germany) and used as templates to be joined together by further PCR with the two primers of pNEO vector. Amplified DNA fragments were digested with restriction enzymes AgeI and KpnI and then ligated into digested pNEO vector. All constructs were verified by sequencing.

#### Protein production

Protein production was as described in Zhou et al. (2021). Briefly, plasmids encoding proteins were transiently expressed in HEK293T (ATCC CRL-11268) cells. The conditioned medium was dialysed and purified with a 5 mL HisTrap nickel column (GE Healthcare) and further polished using a Superdex 75 HiLoad 16/60 gel filtration column (GE Healthcare).

#### Bio-Layer Interferometry

BLI experiments were run on an Octet Red 96e machine (Fortebio). To measure the binding affinity of ACE2 with different RBD variants, each RBD was immobilized onto AR2G biosensors (Fortebio) and serial dilutions of ACE2 were used as analytes. To measure the binding affinity of monoclonal antibodies with RBD variants, each his-tagged RBD was immobilized onto Ni-NTA biosensors (Fortebio) and antibodies (Dejnirattisai et al., 2021a) were used as analytes All experiments were run at 30°C. Data were recorded using software Data Acquisition 11.1 (Fortebio) and Data Analysis HT 11.1 (Fortebio) with a 1:1 fitting model used for the analysis.

#### Antibody production

AstraZeneca and Regeneron antibodies were provided by AstraZeneca, Vir, Lilly and Adagio antibodies were provided by Adagio. For the antibodies heavy and light chains of the indicated antibodies were transiently transfected into 293Y cells and antibody purified from supernatant on protein A.

#### Crystallization

WT RBD was mixed with 222 Fab and 278 Fab, L452R mutant RBD was mixed with 75 Fab and 253 Fab, and T478K mutant RBD was mixed with 45 Fab and 253 Fab in a 1:1:1 molar ratio to a final concentration of 7.0 mg ml^−1^. All samples were incubated at room temperature for 30 min. Crystallization experiments were set up with a Cartesian Robot in Crystalquick 96-well X plates (Greiner Bio-One) using the nanoliter sitting-drop vapor-diffusion method, with 100 nL of protein plus 100 nL of reservoir in each drop, as previously described (Walter et al., 2003). Good crystals of WT RBD/222-278 Fab complex were obtained from Molecular Dimensions Morpheus condition H1, containing 0.1 M amino acids (Glu, Ala, Gly, Lys, Ser), 0.1 M MES/imidazole pH 6.5, 10% (w/v) PEG 20000 and 20% (v/v) PEG MME 550. Good crystals of L452R mutant RBD/75-253 complex were obtained from Hampton Research PEGRx condition 44, containing 0.1 M BIS-TRIS pH 6.5 and 16% (w/v) PEG 10000. Crystals of T478K mutant RBD/45-253 complex were obtained from Hampton Research PEGRx condition 45, containing 0.1 M BICINE pH 8.5 and 20% (w/v) PEG 10,000.

#### X-ray data collection, structure determination and refinement

Crystals of WT RBD/222-278 Fab complex were mounted in loops and frozen by directly dipping in liquid nitrogen. Crystals of L452R mutant RBD/75-253 and T478K mutant RBD/45-253 complexes were mounted and dipped in solution containing 25% glycerol and 75% mother liquor for a second before being frozen in liquid nitrogen. Diffraction data were collected at 100 K at beamline I03 of Diamond Light Source, UK. All data were collected as part of an automated queue system allowing unattended automated data collection (https://www.diamond.ac.uk/Instruments/Mx/I03/I03-Manual/Unattended-Data-Collections.html). Diffraction images of 0.1° rotation were recorded on an Eiger2 XE 16M detector (exposure time of either 0.006 or 0.009 s per image, beam size 80 × 20 μm, 100% beam transmission and wavelength of 0.9763 Å). Data were indexed, integrated and scaled with the automated data processing program Xia2-dials (Winter, 2010; Winter et al., 2018). Data of 360° was collected from a frozen crystal for each of the WT RBD/222-278 and T478K-RBD/45-253 Fab complexes. Dataset of L452R-RBD/75-253 were merged from four crystals (360° from each crystal).

Structures were determined by molecular replacement with PHASER (McCoy et al., 2007) using search models of SARS-CoV-2 RBD-EY6A-222 (PDB ID 7NX6) (Dejnirattisai et al., 2021b) for RBD/222-278 complex, RBD/75-253 (PDB ID, 7BEN) (Dejnirattisai et al., 2021a) for L452R-RBD/75-253 complex, and RBD/45-88 (PDB ID, 7BEL) and RBD/75-253 (PDB ID, 7BEN) (Dejnirattisai et al., 2021a). Model rebuilding with COOT (Emsley and Cowtan, 2004) and refinement with PHENIX (Liebschner et al., 2019) were done for all the structures. There is one ternary complex in the asymmetric unit of RBD/222-278 crystal, and two complexes in the asymmetric unit of both L452R-RBD/75-253 and T478K-RBD/45-253 crystals. The ChCl domains of Fab 45 in the T478K-RBD/45-253 complex are flexible and have poor electron density. Data collection and structure refinement statistics are given in Table S2. Structural comparisons used SHP (Stuart et al., 1979), residues forming the RBD/Fab interface were identified with PISA (Krissinel and Henrick, 2007) and figures were prepared with PyMOL (The PyMOL Molecular Graphics System, Version 1.2r3pre, Schrödinger, LLC).

#### Antigenic space plots

Log of IC_50_ values for each serum/virus strain pair were assembled into vectors for each virus strain. 113 sera from a range of natural infections and vaccinations were used in total and compared against 7 virus strains, assembling a 113x7 matrix. Single value decomposition of this serum/virus strain pair matrix was carried out, producing weighted orthogonal vectors representing the axes of variation within the data and each strain was expressed as a vector in this new orthogonal basis. The largest axis of variation was largely identical for each strain, representing the positivity in common with all log dilution values. The 2nd, 3rd and 4th major axes were plotted using cluster4x (Ginn, 2020) to show the separation between each virus strain in antigenic space.

### Quantification and statistical analysis

Statistical analyses are reported in the results and figure legends. Neutralization was measured by FRNT. The percentage of focus reduction was calculated and IC_50_ was determined using the probit program from the SPSS package. The Wilcoxon matched-pairs signed rank test was used for the analysis and two-tailed P values were calculated and geometric mean values. BLI data were analyzed using Data Analysis HT 11.1 (Fortebio) with a 1:1 fitting model.
