# Supplementary material for: Reduced neutralization of SARS-CoV-2 B.1.617 by vaccine and convalescent serum
Source: Cell. 2021 Aug 5;184(16):4220–4236.e13. doi: 10.1016/j.cell.2021.06.020 (PMC8218332; doi:10.1016/j.cell.2021.06.020)
Supplement: Document S1. Tables S1–S5 [file mmc1.pdf]

Cell, Volume *184*

Supplemental information

Reduced neutralization of SARS-CoV-2 B.1.617

by vaccine and convalescent serum

Chang Liu, Helen M. Ginn, Wanwisa Dejnirattisai, Piyada Supasa, Beibei Wang, Aekkachai Tuekprakhon, Rungtiwa Nutalai, Daming Zhou, Alexander J. Mentzer, Yuguang Zhao, Helen M.E. Duyvesteyn, César López-Camacho, Jose Slon-Campos, Thomas S. Walter, Donal Skelly, Sile Ann Johnson, Thomas G. Ritter, Chris Mason, Sue Ann Costa Clemens, Felipe Gomes Naveca, Valdinete Nascimento, Fernanda Nascimento, Cristiano Fernandes da Costa, Paola Cristina Resende, Alex Pauvolid-Correa, Marilda M. Siqueira, Christina Dold, Nigel Temperton, Tao Dong, Andrew J. Pollard, Julian C. Knight, Derrick Crook, Teresa Lambe, Elizabeth Clutterbuck, Sagida Bibi, Amy Flaxman, Mustapha Bittaye, Sandra Belij-Rammerstorfer, Sarah C. Gilbert, Tariq Malik, Miles W. Carroll, Paul Klenerman, Eleanor Barnes, Susanna J. Dunachie, Vicky Baillie, Natali Serafin, Zanele Ditse, Kelly Da Silva, Neil G. Paterson, Mark A. Williams, David R. Hall, Shabir Madhi, Marta C. Nunes, Philip Goulder, Elizabeth E. Fry, Juthathip Mongkolsapaya, Jingshan Ren, David I. Stuart, and Gavin R. Screaton

Table S1. NT50 titres against pseudo typed lentiviruses expressing full-length spike of SARS-CoV-2 strains Victoria, B.1.617.1 , B.1.617.2, B.1.1.519 and B.1.429, live virus strains Victoria and B.1.617.2, and KD values for binding to different RBDs by BLI. (A) 20 human monoclonal antibodies (B) 11 human monoclonal antibodies in clinical trials. The data underpinning the Victoria live virus neutralization curves have been previously reported (Supasa et al, 2021). Related to Figure2.

mAb	IC50 (ug/ml)							FRNT50 ratio										Immunoglobulin gene usage		
	Pseudovirus					Authentic virus		Pseudovirus				Authentic virus	KD (nM)							
	Victoria	B.1.617.1	B.1.617.2	B.1.1.519	B.1.429	Victoria	B.1.617.2	B.1.617.1 /Victoria	B.1.617.2 /Victoria	B.1.1.519 /Victoria	B.1.429/ Victoria	B.1.617.2/ Victoria	Victor-ia RBD	RBD L452R	RBD T478K	RBD L452R, E484Q	RBD L452R, T478K	IGHV	K/λ	IGLV
40	0.008 ± 0.000	0.024 ± 0.008	0.024±0.002	0.017±0.002	0.011±0.000	0.026 ± 0.007	0.029 ± 0.010	3.0	2.8	2.1	1.4	1.1	0.34	0.50	0.16	0.33	0.47	3-66	K	1-33 or 1D-33
55	0.019 ± 0.002	0.010 ± 0.004	0.007±0.002	0.014±0.002	0.005±0.001	0.095 ± 0.015	0.016 ± 0.005	0.5	0.3	0.7	0.3	0.2	0.10	0.13	0.29	0.30	0.23	1-58	K	3-20
58	0.032 ± 0.005	0.690 ± 0.313	1.005±0.017	0.032±0.002	0.497±0.049	0.041 ± 0.003	6.434 ± 2.623	21.6	26.2	1.0	15.5	156.3	0.14	0.50	0.19	1.5	0.42	3-9	λ	3-21
88	0.010 ± 0.002	0.072 ± 0.013	0.046±0.009	0.026±0.005	0.015±0.001	0.033 ± 0.001	0.039 ± 0.007	7.2	3.8	2.6	1.5	1.2	4.4	2.7	2.4	1.9	5.8	4-61	λ	1-36
132	0.020 ± 0.003	0.048 ± 0.005	0.043±0.013	0.038±0.011	0.022±0.022	0.048 ± 0.000	0.051 ± 0.013	2.4	1.6	1.9	1.1	1.1	2.7	3.6	3.2	0.31	7.3	4-34	λ	7-46
150	0.011 ± 0.004	0.008 ± 0.002	0.007±0.002	0.007±0.002	0.005±0.001	0.012 ± 0.000	0.020 ± 0.001	0.7	0.5	0.6	0.5	1.7	0.57	1.6	1.1	0.70	0.77	3-53	K	1-9
158	0.016 ± 0.005	0.009 ± 0.001	0.021±0.004	0.011±0.002	0.013±0.001	0.031 ± 0.004	0.026 ± 0.002	0.6	1.1	0.7	0.8	0.8	1.4	1.3	0.75	1.4	2.4	3-53	K	1-9
159	>10	>10	>10	0.038±0.003	>10	0.011 ± 0.000	>10	N/A	N/A	<0.004	N/A	>928.4	N/A	N/A	N/A	N/A	N/A	3-30	K	3-20
165	0.009 ± 0.000	0.005 ± 0.001	0.009±0.001	0.013±0.002	0.002±0.001	0.034 ± 0.004	0.027 ± 0.006	0.6	1.0	1.4	0.2	0.8	0.34	0.71	0.51	0.54	1.7	1-58	K	3-20
170	0.024 ± 0.017	>10	0.493±0.047	0.012±0.003	0.550±0.204	0.025 ± 0.004	0.841 ± 0.103	>416.7	22.5	0.5	22.9	33.8	1.0	1.8	1.2	3987	2.6	5-51	K	2D-29
175	0.012 ± 0.001	0.007 ± 0.001	0.005±0.001	0.004±0.001	0.005±0.001	0.026 ± 0.000	0.017 ± 0.003	0.6	0.4	0.3	0.4	0.7	0.33	0.99	0.55	1.7	0.50	3-53	K	1-33 or 1D-33
222	0.010 ± 0.000	0.011 ± 0.001	0.015±0.003	0.007±0.001	0.007±0.000	0.019 ± 0.000	0.018 ± 0.001	1.1	1.2	0.7	0.7	0.9	0.25	1.5	0.20	1.4	0.52	3-53	K	3-20
253	0.012 ± 0.002	0.006 ± 0.002	0.003±0.001	0.006±0.002	0.003±0.001	0.055 ± 0.008	0.005 ± 0.001	0.5	0.3	0.5	0.3	0.1	0.51	1.6	0.78	1.9	0.96	1-58	K	3-20
269	0.004 ± 0.001	0.008 ± 0.001	0.009±0.003	0.012±0.004	0.0040.002	0.030 ± 0.000	0.021 ± 0.004	2.0	1.5	3.0	1.0	0.7	0.52	0.76	0.62	1.5	0.73	3-53	K	1-9
278	0.008 ± 0.004	>10	>10.00	0.005±0.001	4.527±0.747	0.014 ± 0.007	7.374 ± 1.397	>1250.0	>1250.0	0.6	565.9	539.2	1.6	6.3	1.5	22.0	6.4	1-18	K	1-39 or 1D-39
281	0.002 ± 0.000	>10	0.919±0.017	0.002±0.000	0.319±0.016	0.005 ± 0.001	1.494 ± 0.302	>5000.0	451.5	1.0	159.5	302.7	0.33	0.75	0.35	knocked out	0.65	3-7	K	2-24
316	0.003 ± 0.002	>10	0.003±0.001	0.004±0.001	0.004±0.002	0.018 ± 0.007	0.008 ± 0.001	>3333.3	1.3	1.3	0.7	0.4	0.38	1.0	0.69	1623	0.78	1-2	λ	2-8
318	0.018 ± 0.000	0.016 ± 0.007	0.020±0.006	0.008±0.003	0.016±0.011	0.029 ± 0.008	0.018 ± 0.003	0.9	0.8	0.4	1.5	0.6	3.5	11.4	2.8	2.0	5.6	1-58	K	3-20
384	0.002 ± 0.000	>10	0.070±0.015	0.001±0.000	0.009±0.001	0.004 ± 0.001	0.108 ± 0.035	>5000.0	27.5	0.5	4.5	25.8	0.65	0.98	0.84	knocked out	1.9	3-11	K	1-27
398	0.052 ± 0.024	>10	0.133±0.004	0.054±0.004	0.015±0.003	0.091 ± 0.004	0.237 ± 0.038	>192.3	2.5	1.0	0.3	2.6	1.1	7.3	5.5	1793	5.6	3-66	λ	2-8
AZD1061	0.007 ± 0.003	0.033 ± 0.020	N/A	0.004±0.001	N/A	0.013 ± 0.003	0.038 ± 0.006	5.1	N/A	0.6	N/A	2.9	3.9	6.7	3.8	6.7	6.9	N/A	N/A	N/A
AZD8895	0.003 ± 0.002	0.002 ± 0.011	N/A	0.001±0.000	N/A	0.005 ± 0.001	0.003 ± 0.000	0.7	N/A	0.3	N/A	0.5	1.00	2.1	3.7	3.9	1.5	N/A	N/A	N/A
AZD7442	0.002 ± 0.000	0.004 ± 0.002	N/A	0.002±0.000	N/A	0.009 ± 0.000	0.005 ± 0.000	2.0	N/A	1.0	N/A	0.6	2.0	13.0	6.3	2.2	2.4	N/A	N/A	N/A
REGN10987	0.014 ± 0.001	0.021 ± 0.011	N/A	0.008±0.000	N/A	0.032 ± 0.007	0.017 ± 0.009	1.5	N/A	0.6	N/A	0.5	0.38	0.36	0.35	0.13	0.38	N/A	N/A	N/A
REGN10933	0.002 ± 0.001	0.010 ± 0.007	N/A	0.001±0.000	N/A	0.004 ± 0.002	0.003 ± 0.001	5.0	N/A	0.5	N/A	0.6	0.81	2.2	1.8	0.44	0.74	N/A	N/A	N/A
ADG10	0.007 ± 0.004	0.009 ± 0.004	N/A	0.011±0.001	N/A	0.006 ± 0.000	0.026 ± 0.005	1.3	N/A	1.6	N/A	4.6	N/A	N/A	N/A	N/A	N/A	N/A	N/A	N/A
ADG20	0.002 ± 0.000	0.005 ± 0.001	N/A	0.003±0.001	N/A	0.004 ± 0.001	0.006 ± 0.001	2.5	N/A	1.5	N/A	1.4	N/A	N/A	N/A	N/A	N/A	N/A	N/A	N/A
ADG30	0.014 ± 0.000	0.048 ± 0.015	N/A	0.016±0.002	N/A	0.007 ± 0.002	0.033 ± 0.007	3.4	N/A	1.1	N/A	5.0	N/A	N/A	N/A	N/A	N/A	N/A	N/A	N/A
LY-CoV555	0.004 ± 0.001	>10	N/A	0.005±0.001	N/A	0.006 ± 0.002	8.311 ± 4.059	>2500.0	N/A	1.3	N/A	1284.3	N/A	N/A	N/A	N/A	N/A	N/A	N/A	N/A
LY-CoV16	0.026 ± 0.006	0.010 ± 0.001	N/A	0.006±0.001	N/A	0.034 ± 0.007	0.012 ± 0.002	0.4	N/A	0.2	N/A	0.4	N/A	N/A	N/A	N/A	N/A	N/A	N/A	N/A
S309	>10	0.238 ± 0.051	N/A	0.078±0.015	N/A	0.040 ± 0.005	0.113 ± 0.028	<0.024	N/A	<0.008	N/A	2.8	N/A	N/A	N/A	N/A	N/A	N/A	N/A	N/A

Table S2 Data collection and refinement statistics of RBD complexes. Related to X-ray data collection, structure determination and refinement, STAR Methods

[a] Values in parentheses are for highest-resolution shell.

Structure	RBD/222-278	L452R-RBD/75-253	T478K-RBD/45-253
PDB ID	7OR9	7ORB	7ORA
Data collection			
Space group	$P2_12_12_1$	$P2_1$	$P2_1$
Cell dimensions			
a, b, c (Å)	70.7, 114.5, 177.9	93.2, 149.4, 115.0	51.6, 182.4, 142.4
a, b, g (°)	90, 90, 90	90, 92.0, 90	90, 93.0, 90
Resolution (Å)	70–2.34 (2.38–2.34)[a]	75–2.50 (2.54–2.50)	61–2.60 (2.64–2.60)
R_{merge}	0.100 (---)	0.300 (---)	0.245 (---)
R_{pim}	0.030 (0.806)	0.058 (0.386)	0.101 (0.860)
I/s(I)	16.8 (0.8)	5.6 (0.4)	7.3 (0.6)
$CC_{1/2}$	0.999 (0.348)	0.993 (0.609)	0.986 (0.302)
Completeness (%)	86.2 (47.5)	99.8 (94.6)	90.5 (49.0)
Redundancy	11.6 (5.2)	28.1 (26.4)	6.7 (6.6)
Refinement			
Resolution (Å)	57–2.34	74–2.50	52–2.60
No. reflections	50501/2653	102853/5504	68969/3634
R_{work} / R_{free}	0.197/0.233	0.212/0.252	0.208/0.249
No. atoms			
Protein	8070	16156	16276
Ligand/ion/water	216	808	390
B factors (Å^2)			
Protein	61	64	66
Ligand/ion/water	67	73	57
r.m.s. deviations			
Bond lengths (Å)	0.002	0.003	0.002
Bond angles (°)	0.6	0.6	0.6

Table S3. NT50 titres against pseudo typed lentiviruses expressing full-length spike of SARS-CoV-2 strains Victoria and B.1.617.1, and live virus strains Victoria and B.1.617.2 (A) 34 convalescent plasma during early pandemic in UK (B) plasma from 18 patients infected with B.1.1.7 (C) serum from 14 patients infected with B.1.351 (D) serum from 17 patients infected with P.1 . The data underpinning the Victoria live virus neutralization curves of convalescent samples have been previously reported (Supasa et al, 2021). Related to Figure5 and 6.

		NT50 (Reciprocal plasma dilution)				NT50 ratio	
		Pseudo virus		Authentic virus			
		Victoria	B.1.617.1	Victoria	B.1.617.2	Victoria/B.1.67 1.1	Victoria/B.1.61 7.2
**Early pandemic UK**	Convalescent 1	99	<20	61	<20	>5.0	>3.1
	Convalescent 2	1098	165	689	41	6.7	16.6
	Convalescent 3	677	677	526	231	1.0	2.3
	Convalescent 4	992	817	409	445	1.2	0.9
	Convalescent 5	1016	54	369	136	18.8	2.7
	Convalescent 6	1417	820	1270	460	1.7	2.8
	Convalescent 7	716	115	274	124	6.2	2.2
	Convalescent 8	474	288	633	360	1.6	1.8
	Convalescent 9	N/A	N/A	667	81	N/A	8.2
	Convalescent 10	N/A	N/A	124	<20	N/A	>6.2
	Convalescent 11	N/A	N/A	102	127	N/A	0.8
	Convalescent 12	585	97	339	95	6.0	3.6
	Convalescent 13	362	214	331	55	1.7	6.0
	Convalescent 14	514	52	438	465	9.9	0.9
	Convalescent 15	8300	7086	6397	2916	1.2	2.2
	Convalescent 16	124	<20	44	<20	>6.2	>2.2
	Convalescent 17	1188	33	1115	239	35.6	4.7
	Convalescent 18	480	85	242	71	5.7	3.4
	Convalescent 19	78	19	29	<20	4.2	>1.5
	Convalescent 20	552	204	154	60	2.7	2.6
	Convalescent 21	894	135	487	76	6.6	6.4
	Convalescent 22	1596	497	438	236	3.2	1.9
	Convalescent 23	661	135	381	189	4.9	2.0
	Convalescent 24	2976	405	1647	900	7.4	1.8
	Convalescent 25	2609	750	913	747	3.5	1.2
	Convalescent 26	1677	424	1880	832	4.0	2.3
	Convalescent 27	1664	227	1464	829	7.3	1.8
	Convalescent 28	652	333	361	621	2.0	0.6
	Convalescent 29	3117	982	2859	1143	3.2	2.5
	Convalescent 30	1341	447	1109	425	3.0	2.6
	Convalescent 31	1518	454	811	556	3.3	1.5
	Convalescent 32	764	567	395	317	1.3	1.2
	Convalescent 33	N/A	N/A	1144	302	N/A	3.8
	Convalescent 34	1289	830	676	150	1.6	4.5
**B.1.1.7**	B.1.1.7 P3	101	27	143	39	3.7	3.7
	B.1.1.7-P4	22442	15436	88889	109481	1.5	0.8
	B.1.1.7 P5	1423	107	1839	384	13.3	4.8
	B.1.1.7 P6	655	69	562	54	9.5	10.4
	B.1.1.7 P7	2073	1623	2936	3049	1.3	1.0
	B.1.1.7 P8	2459	749	4696	535	3.3	8.8
	B.1.1.7 P9	2046	145	1777	533	14.1	3.3
	B.1.1.7 P10	3019	373	2484	1182	8.1	2.1
	B.1.1.7 P11	3243	205	1632	532	15.8	3.1
	B.1.1.7 P14	1444	665	1555	911	2.2	1.7
	B.1.1.7 P15	174	79	227	778	2.2	0.3
	B.1.1.7-P16	1463	292	2902	2016	5.0	1.4
	B.1.1.7-P17	803	415	3781	566	1.9	6.7
	B.1.1.7-P18	990	466	2641	732	2.1	3.6
	B.1.1.7-P19	575	54	775	211	10.7	3.7
	B.1.1.7-P20	1647	1197	2614	1509	1.4	1.7
	B.1.1.7-P22	422	172	390	182	2.4	2.1
	B.1.1.7-P23	2243	504	864	185	4.4	4.7
**B.1.351**	SA01	586	402	379	214	1.5	1.8
	SA02	564	876	597	629	0.6	0.9
	SA03	107	18	71	<20	5.9	>3.55
	SA04	824	1231	240	293	0.7	0.8
	SA05	166	27	67	<20	6.1	>3.35
	SA06	295	121	69	<20	2.4	>3.45
	SA07	1384	508	463	374	2.7	1.2
	SA08	360	239	641	30	1.5	21.7
	SA09	584	259	888	<20	2.3	>44.4
	SA10	57	98	80	<20	0.6	>4.4
	SA11	530	379	1234	83	1.4	14.9
	SA12	1026	1142	2148	390	0.9	5.5
	SA13	53	10	268	<20	5.1	>13.4
	SA14	678	511	595	123	1.3	4.8
**P.1**	P.1-1	274	125	86	<20	2.2	>4.3
	P.1-2	21	22	<20	<20	1.0	N/A
	P.1-3	108	58	20	<20	1.9	N/A
	P.1-4	152	116	29	<20	1.3	>1.4
	P.1-5	56	31	<20	<20	1.8	N/A
	P.1-6	1751	1039	2505	1311	1.7	1.9
	P.1-7	313	12	123	<20	25.1	>6.1
	P.1-8	151	100	40	<20	1.5	>2.0
	P.1-9	111	107	23	<20	1.0	>1.2
	P.1-10	107	19	<20	<20	5.5	N/A
	P.1-11	157	82	123	<20	1.9	>6.2
	P.1-12	62	48	<20	<20	1.3	N/A
	P.1-13	202	87	<20	<20	2.3	N/A
	P.1-14	121	39	87	<20	3.1	>4.3
	P.1-15	1555	2050	8199	2551	0.8	3.2
	P.1-16	337	194	828	333	1.7	2.5
	P.1-17	1489	364	1654	227	4.1	7.3

Table S4. NT50 titres against pseudo typed lentiviruses expressing full-length spike of SARS-CoV-2 strains Victoria and B.1.617.1, and strains Victoria and B.1.617.2 (A) Serum from 25 recipients of Pfizer-BioNTech vaccine. (B) Oxford-AstraZeneca vaccine. The data underpinning the Victoria live virus neutralization curves have been previously reported (Supasa et al, 2021). Related to Figure7.

	Day Post-boost	NT50 (Reciprocal plasma dilution)				NT50 ratio	
		Pseudovirus		Authentic virus			
		Victoria	B.1.617.1	Victoria	B.1.617.2	Victoria/B.1.671.1	Victoria/B.1.617.2
Pfizer1	7	1575	790	1149	540	2.0	2.1
Pfizer2	7	82	10	10	10	8.2	1.0
Pfizer3	7	1812	686	1727	644	2.6	2.7
Pfizer4	8	4675	3179	2234	3604	1.5	0.6
Pfizer5	7	3239	999	3016	992	3.2	3.0
Pfizer6	7	1994	1208	1521	875	1.7	1.7
Pfizer7	7	1466	973	609	636	1.5	1.0
Pfizer8	7	3115	953	4340	726	3.3	6.0
Pfizer9	7	888	405	1467	217	2.2	6.8
Pfizer10	7	2315	493	1757	338	4.7	5.2
Pfizer11	7	1418	307	860	138	4.6	6.2
Pfizer12	7	1643	619	1749	964	2.7	1.8
Pfizer13	7	2744	937	1851	932	2.9	2.0
Pfizer14	7	584	216	407	177	2.7	2.3
Pfizer15	8	1823	491	1285	466	3.7	2.8
Pfizer16	8	1094	216	1286	336	5.1	3.8
Pfizer17	8	2358	558	1810	811	4.2	2.2
Pfizer18	8	1872	1227	1198	474	1.5	2.5
Pfizer19	8	983	603	466	155	1.6	3.0
Pfizer20	8	1603	1156	1539	502	1.4	3.1
Pfizer21	9	347	34	184	39	10.3	4.7
Pfizer22	11	678	455	1061	586	1.5	1.8
Pfizer23	12	1149	297	1658	365	3.9	4.5
Pfizer24	12	1082	558	1155	1036	1.9	1.1
Pfizer25	15	3496	2494	8092	4256	1.4	1.9
AstraZeneca 1	28	489	333	495	235	1.5	2.1
AstraZeneca 2	28	440	345	580	308	1.3	1.9
AstraZeneca 3	28	90	12	253	10	7.2	25.3
AstraZeneca 4	28	349	311	183	101	1.1	1.8
AstraZeneca 5	28	681	56	432	121	12.2	3.6
AstraZeneca 6	28	169	30	764	134	5.6	5.7
AstraZeneca 7	28	228	127	133	10	1.8	13.3
AstraZeneca 8	28	333	81	257	57	4.1	4.5
AstraZeneca 9	28	134	106	501	194	1.3	2.6
AstraZeneca 10	28	93	133	357	217	0.7	1.6
AstraZeneca 11	14	192	116	334	90	1.7	3.7
AstraZeneca 12	14	88	51	250	54	1.7	4.6
AstraZeneca 13	14	373	218	122	26	1.7	4.8
AstraZeneca 14	14	188	91	212	41	2.1	5.1
AstraZeneca 15	14	572	87	789	69	6.6	11.5
AstraZeneca 16	14	202	156	538	201	1.3	2.7
AstraZeneca 17	14	763	208	1159	184	3.7	6.3
AstraZeneca 18	14	233	102	353	122	2.3	2.9
AstraZeneca 19	14	1031	443	975	224	2.3	4.3
AstraZeneca 20	14	188	64	169	30	2.9	5.7
AstraZeneca 21	14	224	49	155	29	4.5	5.4
AstraZeneca 22	14	367	49	152	39	7.6	3.9
AstraZeneca 23	14	96	43	126	27	2.3	4.7
AstraZeneca 24	14	612	102	293	223	6.0	1.3
AstraZeneca 25	14	67	32	94	10	2.1	9.4

Table S5. Primer sequences used to generate Pseudoviruses and RBD mutants. Related to Plasmid construction and pseudotyped lentiviral particles production, STAR METHODS.

Primer	Sequence (5′ to 3′)
**Victoria insert fragments**	
S247R_F	GCTGGCCCTGCACAGAAGATATCTTACACCAGGC
S247R_R	GCCTGGTGTAAGATATCTTCTGTGCAGGGCCAG
**B.1.617.1 insert fragments**	
E154K_F	CAACAAGAGCTGGATGAAGAGCGAGTTCCGCG
E154K_R	CGCGGAACTCGCTCTTCATCCAGCTCTTGTTG
L452R_F	GGAGGCAATTACAATTACCGGTACAGACTGTTCAGAAAG
L452R_R	CTTTCTGAACAGTCTGTACCGGTAATTGTAATTGCCTCC
E484Q_F	CCGTGTAATGGCGTGCAGGGCTTCAATTGCTAC
E484Q_R	GTAGCAATTGAAGCCCTGCACGCCATTACACGG
D614G_F	CGTGCTGTACCAGGGCGTGAATTGCACCG
D614G_R	CGGTGCAATTCACGCCCTGGTACAGCACG
P681R_F	CCCAGACCAATAGCCGTAGAAGAGCCAGAAG
P681R_R	CTTCTGGCTCTTCTACGGCTATTGGTCTGGG
E1072K/K1073R_F	CTACGTGCCTGCCCAGAAGAGGAATTTCACCACCGC
E1072K/K1073R_R	GCGGTGGTGAAATTCCTCTTCTGGGCAGGCACGTAG
T95I_F	GCGTGTACTTCGCCAGCATCGAGAAGAGCAATATC
T95I_R	GATATTGCTCTTCTCGATGCTGGCGAAGTACACGC
G142D_F	GTTCTGCAATGACCCTTTCCTGGATGTTTATTATCATAAGAACAAC
G142D_R	GTTGTTCTTATGATAATAAACATCCAGGAAAGGGTCATTGCAGAAC
Q1071H_F	CCTACGTGCCTGCCCATGAGAAGAATTTCACCA
Q1071H_R	TGGTGAAATTCTTCTCATGGGCAGGCACGTAGG
**B.1.617.2 insert fragments**	
T19R_F	GCAGCCAGTGCGTGAATCTGAGGACCAGAACCCAG
T19R_R	CTGGGTTCTGGTCCTCAGATTCACGCACTGGCTGC
Del156-158_F	CAAGAGCTGGATGGAGAGCGTATATTCGTCGGCTAATAATTGCCC
Del156-158_R	GGGCAATTATTAGCCGACGAATATACGCTCTCCATCCAGCTCTTG
D950N_F	CTGGGCAAGCTGCAGAACGTGGTGAATCAGAATG
D950N_R	CATTCTGATTCACCACGTTCTGCAGCTTGCCCAG
**B.1.1.519 insert fragments**	
T478K_F	TACCAGGCCGGCAGCAAACCGTGTAATGG
T478K_R	CCATTACACGGTTTGCTGCCGGCCTGGTA
T732A_F	GAAATATTACCAGTCTCCATGGCCAAGACCAGCGTGG
T732A_R	CCACGCTGGTCTTGGCCATGGAGACTGGTAATATTTC
**B.1.429 insert fragments**	
S13I_F	CTGCCTCTGGTGAGCATCCAGTGCGTGAATC
S13I_R	GATTCACGCACTGGATGCTCACCAGAGGCAG
W152C_F	CATAAGAACAACAAGAGCTGCATGGAGAGCGAGTTCC
W152C_R	GGAACTCGCTCTCCATGCAGCTCTTGTTGTTCTTATG
P681H_F	ACCCAGACCAATAGCCATAGAAGAGCCAGAAGC
P681H_R	GCTTCTGGCTCTTCTATGGCTATTGGTCTGGGT
**pcDNA3.1 vector**	
pcDNA3.1_BamHI_F	GGATCCATGTTCCTGCTGACCACCAAGAG
pcDNA3.1_Tag_S_EcoRI_R	GAATTCTCACTTCTCGAACTGAGGGTGGC
pcDNA3.1_Tag_S_EcoRI_F	GCCACCCTCAGTTCGAGAAGTGAGAATTC
pcDNA3.1_BamHI_R	CTCTTGGTGGTCAGCAGGAACATGGATCC
**pNEO vector**	
PV_RBD_F	TGATGGGTTGCGTAGCTGAAACCGGTCACCATCACCATCACCATACCAATCTGTGCCCTTTCGGCGAGGTGTTC
PV_RBD_R	CTGGAACAGCACCTCCAGGGTACCTCACTTTTTGCCGCACACGGTAGCGGGAGC
pNEO_F	GCTGGTTGTTGTGCTGTCTCATC
pNEO_R	CGTAAAAGGAGCAACATAG
